# MAGs-centric crack: how long will, spore-positive *Frankia* and most *Protofrankia*, microsymbionts remain recalcitrant to axenic growth?

**DOI:** 10.3389/fmicb.2024.1367490

**Published:** 2024-07-31

**Authors:** Maher Gtari, Radhi Maaoui, Faten Ghodhbane-Gtari, Karim Ben Slama, Imed Sbissi

**Affiliations:** ^1^Department of Biological and Chemical Engineering, USCR Molecular Bacteriology and Genomics, National Institute of Applied Sciences and Technology, University of Carthage, Tunis, Tunisia; ^2^Higher Institute of Biotechnology Sidi Thabet, University of La Manouba, Tunisia; ^3^LR Bioresources, Environment, and Biotechnology (LR22ES04), Higher Institute of Applied Biological Sciences of Tunis, University of Tunis El Manar, Tunis, Tunisia; ^4^LR Pastoral Ecology, Arid Regions Institute, University of Gabes, Medenine, Tunisia

**Keywords:** *Frankia*, *Protofrankia*, obligate/facultative microsymbionts, metagenome-assembled genomes, ecological traits, axenic conditions, phenotype predictive tools, trait-based model

## Abstract

Nearly 50 years after the ground-breaking isolation of the primary *Comptonia peregrina* microsymbiont under axenic conditions, efforts to isolate a substantial number of *Protofrankia* and *Frankia* strains continue with enduring challenges and complexities. This study aimed to streamline genomic insights through comparative and predictive tools to extract traits crucial for isolating specific *Frankia* in axenic conditions. Pangenome analysis unveiled significant genetic diversity, suggesting untapped potential for cultivation strategies. Shared metabolic strategies in cellular components, central metabolic pathways, and resource acquisition traits offered promising avenues for cultivation. Ecological trait extraction indicated that most uncultured strains exhibit no apparent barriers to axenic growth. Despite ongoing challenges, potential caveats, and errors that could bias predictive analyses, this study provides a nuanced perspective. It highlights potential breakthroughs and guides refined cultivation strategies for these yet-uncultured strains. We advocate for tailored media formulations enriched with simple carbon sources in aerobic environments, with atmospheric nitrogen optionally sufficient to minimize contamination risks. Temperature adjustments should align with strain preferences—28–29°C for *Frankia* and 32–35°C for *Protofrankia*—while maintaining an alkaline pH. Given potential extended incubation periods (predicted doubling times ranging from 3.26 to 9.60 days, possibly up to 21.98 days), patience and rigorous contamination monitoring are crucial for optimizing cultivation conditions.

## Introduction

Actinobacterial taxa within the four genera - *Frankia*, *Protofrankia*, *Parafrankia*, and *Pseudofrankia*, of the family *Frankiaceae* (Gtari, 2022), colonize the nitrogen-fixing root nodules in actinorhizal plants, mainly engaging in mutualistic symbiosis (Huss-Danell, 1997). Initially classified as obligate microsymbionts with elusive identities (Becking, 1970), efforts to isolate and characterize these microsymbionts faced challenges such as slow growth rates, undefined media, and contamination issues (Wheeler et al., 2008). A pivotal breakthrough in 1978 led to the first axenic cultivation of a *Frankia* strain from *Comptonia peregrina* nodules (Callaham et al., 1978). Despite ongoing successful isolations, there has been limited success in culturing *Protofrankia* strains (Persson et al., 2011, 2015; Gtari et al., 2015; Nguyen et al., 2016, 2019; Gueddou et al., 2019; Berckx et al., 2022). Moreover, *Frankia* strains exhibiting the Sp+ (spore-positive phenotype), which can be distinguished from Sp− (spore-negative phenotype) types that produce fewer or no multilocular sporangia in planta (Schwintzer, 1990; Schwob et al., 2018), have not yet been cultured despite numerous attempts (Normand et al., 2017; Herrera-Belaroussi et al., 2020; Pozzi et al., 2020). These yet-uncultured *Frankia* microsymbionts have been extensively studied using various phylogenetic markers, amplified by PCR directly from DNA extracted from the root nodules of their host species. Key markers include 16S rRNA genes (Nazaret et al., 1991; Simonet et al., 1994), ITS (Internal Transcribed Spacer) rRNA gene regions (Ghodhbane-Gtari et al., 2010), the *gln*A gene (glutamine synthetase), the *dna*A gene (chromosome replication initiator), and the *nif*DK IGS (intergenic spacer between *nif*D and *nif*K genes) (Nouioui et al., 2014). More recently, Metagenome-Assembled Genomes (MAGs) have been employed to study these microsymbionts, uncovering the genetic diversity and functional capabilities of these elusive organisms. Additionally, four candidate species have been defined to accommodate uncultured taxa: *Candidatus* Protofrankia datiscae (Persson et al., 2011) and *Candidatus* Protofrankia californiensis (Normand et al., 2017), as well as *Candidatus* Frankia alpina (Pozzi et al., 2020) and *Candidatus* Frankia nodulisporulans (Herrera-Belaroussi et al., 2020).

Leveraging data from MAGs and single-cell amplified genomes (SAGs), efforts are underway to challenge the perception of certain bacteria as “uncultivable” (Gutleben et al., 2018; Lewis and Ettema, 2019; Lewis et al., 2021; Xie et al., 2021; Liu et al., 2022; Laugier, 2023). Through exploration of diverse culture techniques such as co-cultivation strategies, microfluidics, and synthetic biology, the goal is to establish culturomics as a pivotal complement to metagenomics and single-cell genomics, enhancing our understanding of historically challenging-to-culture microbial communities (Lagier et al., 2012; Nowrotek et al., 2019).

If some of *Frankia* strains are genuinely uncultivable, their exclusive symbiotic lifestyle, distinct from their cultured counterparts, is expected to prompt a relaxation of selection on various metabolic functions that become obsolete within the stress-buffered host cells where the microsymbiont is shielded by host stress responses (Wernegreen, 2015; Lajoie and Parfrey, 2022). Consequently, this could lead to genome streamlining, characterized by a gradual loss of functions from the microsymbiont genome that are also present in the host genome (Moran et al., 2008; Lo et al., 2016). To distill traits pertinent to cultivability, genomic information is organized into a metabolomic blueprint that encapsulates ecological traits (Lajoie and Kembel, 2019; Gtari et al., 2024). This integrative approach, permitted to unveil metabolic profiles, exposing unforeseen requirements and guiding the design of tailored conditions for the growth and isolation of previously uncultured microorganisms (Giovannoni et al., 2014; Lewis et al., 2021). Cultivation strategies span specific growth conditions to innovative methods bridging natural habitats and laboratory settings, including environmental condition replication, dormancy release, co-cultivation, and specialized *in situ* cultivation devices (Riva et al., 2022; Kapinusova et al., 2023; Schultz et al., 2023; Yan et al., 2023). Advanced techniques such as genome editing and adaptive evolution further contribute to narrowing the gap between a microorganism’s native environment and controlled laboratory conditions (Liu et al., 2022; Laugier, 2023). A notable example for *Frankia* research involves the achievement of employing a dual approach, integrating comparative genomics with physiological assays on nodule tissues. This methodology facilitated the axenic cultivation of a previously elusive *Protofrankia* microsymbiont associated with *Coriaria* spp. (Gtari et al., 2015).

The main objective of this study is to delve into the ecological and evolutionary imprints of uncultured *Frankia* genomes. Leveraging comparative genomics and advanced genome predictive tools, we seek to unravel the intricate details of their genome content, organization, and predictive functions that could be instrumental in unlocking their growth potential in axenic cultures. Despite persistent challenges, our findings offer a nuanced perspective, laying the groundwork for potential breakthroughs and steering the development of cultivation strategies.

## Materials and methods

### Genome selection

For this study, a comprehensive set of 30 genomes was obtained from the NCBI database, encompassing diverse strains from the four *Frankia* genera, including both uncultured microsymbionts from *Protofrankia* and *Frankia* genera and their closely related cultivated counterparts (Table 1). As per the Minimum Information about a MAG (MIMAG) standards (Bowers et al., 2017), MAGs are classified as ‘high-quality’ if they exhibit >90% completeness and less than 5% contamination. In this study, the completeness of the used MAGs ranges from 82.45 to 97.39%, and contamination levels range from 0.25 to 5.2%. These values indicate that the MAGs meet the criteria necessary for conducting comparative analyses with isolate-assembled genomes (IAGs), thereby substantiating rigorous downstream functional analysis and ensuring the validity of potential conclusions.

**Table 1:** Genome Assembly and Quality Metrics for Bacterial strains used in the present study.

Organism name	Genome Accession	Axenic culture status	Genome assembled origin	Assembly level	Contig N50	Scaffold N50	Assembly sequencing tech	CheckM marker set	CheckM completeness	CheckM contamination
*Frankia casuarinae* CcI3^T^	CP000249	+	Isolate-assembled Genome	1 Scaffold	5,433,628	5,433,628		*Frankia*	99.99	1.52
*Frankia alni* ACN14aT	CT5732132	+	Isolate-assembled Genome	1 Scaffold	7,497,934	7,497,934		*Frankia*	99.96	0.56
*Frankia torreyi* CpI1^T^	JYFN00000000	+	Isolate-assembled Genome	153 Scaffold	99,379	107,928	Illumina	*Frankia*	99.57	1.9
*Frankia canadensis* ARgP5T	FZMO00000000	+	Isolate-assembled Genome	568 Contig	27,216			*Frankia*	98.87	2.33
*Frankia umida* Ag45/Mut15^T^	JALKFT000000000	+	Isolate-assembled Genome	157 Scaffold	112,473	188,571	Illumina	*Frankia*	91.62	0.5
*Frankia gtarii* Agncl-4^T^	JANEZS000000000	+	Isolate-assembled Genome	442 Contig	38,429		Illumina	*Frankia*	98.86	1.47
*Frankia* sp. QA3	AJWA00000000	+	Isolate-assembled Genome	1 Scaffold	130,116	7,590,853	Illumina	*Frankia*	94.69	1.36
*Candidatus* Frankia nodulisporulans AgUmASt1	CADDZU000000000	−	Metagenome-assembled genome	211 Scaffold	21,949	30,049	Illumina	*Frankia*	82.45	0.86
*Candidatus* Frankia alpina AvVan	SSXH00000000	−	Metagenome-assembled genome	1,228 Contig	6,553		Illumina	*Frankia*	86.66	0.34
*Candidatus* Frankia alpina AiOr	CADCWT000000000	−	Metagenome-assembled genome	669 contig	17,359		Illumina	*Frankia*	87.87	0.38
*Candidatus* Frankia nodulisporulans AgTrS	CADCWS000000000	−	Metagenome-assembled genome	612 Contig	15,284		Illumina	*Frankia*	87.26	0.29
*Candidatus* Frankia nodulisporulans AgUmASH1	CADDZW000000000	−	Metagenome-assembled genome	231 Scaffold	17,387	26,450	Illumina	*Frankia*		
*Parafrankia elaeagni* BMG5.12^T^	ARFH00000000	+	Isolate-assembled genome	135 Scaffold	162,237	162,237	Illumina	*Parafrankia*	99.59	0.19
*Parafrankia irregularis* G2^T^	FAOZ00000000	+	Isolate-assembled genome	83 Scaffold	189,407	227,129		*Parafrankia*	99.64	1.03
*Parafrankia discariae* BCU110501^T^	ARDT00000000	+	Isolate-assembled genome	194 Scaffold	127,450	132,179	Illumina	*Parafrankia*	99.48	0.97
*Parafrankia colletiae* Cc1 17^T^	MBLM00000000	+	Isolate-assembled genome	195 Contig	118,488		Illumina	*Parafrankia*	96.86	2.51
*Parafrankia soli* Cj^T^	MAXA00000000	+	Isolate-assembled genome	289 Contig	88,066		Illumina	*Parafrankia*	95.59	0.89
*Protofrankia coriariae* BMG5.1^T^	JWIO00000000	+	Isolate-assembled genome	116 Scaffold	41,204	105,614	Illumina	*Protofrankia*	88.17	0.76
*Protofrankia* sp. BMG5.30	MOME00000000	+	Isolate assembled genome	94 Contig	124,729		Illumina	*Protofrankia*	98.52	0.44
*Candidatus* Protofrankia datiscae Dg1	CP002801	−	Metagenome-assembled genome	3 Scaffold	5,323,186	5,323,186	Illumina	*Protofrankia*	95.83	0.25
*Protofrankia* symbiont of *Coriaria ruscifolia* Cv1_Ct_nod1	CAAAFR000000000	−	Metagenome-assembled genome	203 Scaffold	32,417	57,864	Illumina	*Protofrankia*	97.39	0.9
*Candidatus* Protofrankia californiensis Dg2	FLUV00000000	−	Metagenome-assembled genome	2,738 Contig	3,103			*Protofrankia*	84.78	5.2
*Protofrankia* symbiont of *Coriaria nepalensis* Dg1_Cn_nod	CAAAHA000000000	−	Metagenome-assembled genome	1,126 Contig	8,186		Illumina	*Protofrankia*	84.78	5.2
*Candidatus* Protofrankia meridionalis Cppng1_Ca_nod	CAAAFQ000000000	−	Metagenome-assembled genome	101 Scaffold	7,881	87,705	Illumina	*Protofrankia*		
*Candidatus* Protofrankia meridionalis Cppng1	CADDZT000000000	−	Metagenome-assembled genome	101 Scaffold	7,881	87,705	Illumina	*Protofrankia*		
*Pseudofrankia inefficax* EuI1c^T^	CP002299	+	Isolate-assembled genome	1 Scaffold	8,815,781	8,815,781	Illumina	*Pseudofrankia*	100	1.36
*Pseudofrankia saprophytica* CN3^T^	AGJN00000000	+	Isolate-assembled genome	2 Scaffold	221,503	8,190,446	Illumina	*Pseudofrankia*	99.95	1.83
*Pseudofrankia asymbiotica* M16386^T^	MOMC00000000	+	Isolate-assembled genome	174 Contig	117,461		Illumina	*Pseudofrankia*	99.85	1.99
*Pseudofrankia* sp. DC12	LANG00000000	+	Isolate-assembled genome	1 Scaffold	837,743	6,884,336	Illumina	*Pseudofrankia*	99.41	1.27
*Pseudofrankia* sp. BMG5.36	MBLO00000000	+	Isolate-assembled genome	280 Scaffold	84,949		Illumina	*Pseudofrankia*	95.39	2.75

* The symbols (+) and (−) indicate the cultivability in axenic conditions: (+) denotes strains isolated in axenic conditions, while (−) indicates strains that have not yet been cultured despite several attempts (Persson et al., 2011, 2015; Nguyen et al., 2016, 2019; Normand et al., 2017; Herrera-Belaroussi et al., 2020; Pozzi et al., 2020; Berckx et al., 2022).

### Genome comparison

Pairwise Mash distances were calculated using Mash v2.1 (Ondov et al., 2016). Digital DNA–DNA hybridization (dDDH) values and confidence intervals were calculated using GGDC version 3.0 (Meier-Kolthoff et al., 2013). Pairwise average nucleotide identity (ANI) were calculated at https://www.ezbiocloud.net (Yoon et al., 2017).

### Genomic evolutionary signatures

For the presence of insertion sequences, ISfinder (Siguier et al., 2006, 2014) was employed. Pseudogene prediction was conducted utilizing Pseudofinder (Syberg-Olsen et al., 2022), and CRISPR elements were identified using the CRISPR Recognition Tool (Bland et al., 2007). Data concerning plasmid-like and virus-like components within a genome were sourced from IMG-M (Chen et al., 2023), incorporating geNomad for the automated identification of both virus-like and plasmid-like entities. Putative horizontal gene transfer (HGT) events were identified using data from IMG-M (Chen et al., 2023).

### Comparative analysis of cultured and uncultured strains

Pangenome analysis which permitted to categorize genes into core genes include strict-core (>99 to 100% prevalence) and soft-core genes (>95 to >96% prevalence), and cloud genes, was performed using Panaroo v1.3.4 (Tonkin-Hill et al., 2020) with default settings (sequence identity threshold 95%, protein family sequence identity threshold 70%, length difference threshold 95%). Gene gain and loss events were assessed using Panstripe (Tonkin-Hill et al., 2023) alongside the phylogenetic tree from the core gene alignment constructed using IQ-TREE (Nguyen et al., 2015), along with the Panaroo gene presence-absence matrix.

To investigate the evolution of gene content, ancestral gene numbers and events were estimated using the Dollo parsimony method implemented in COUNT v9.1106 program, which does not require an out-group species for the phylogenetic tree (Csurös, 2010). The final tree was generated using the web application chiplot (Xie et al., 2023).

To explore genomic differences in uncultured and cultured strains, a detailed comparative analysis was conducted using Orthovenn3 using the OrthoFinder algorithm, with an *e*-value of 1e^−2^ and an inflation value of 1.50, was employed for orthologous analyses and GO term enrichment analysis was also performed using Orthovenn3 (Sun et al., 2023). Pairwise non-synonymous and synonymous substitution ratios (dN/dS) for each single-copy orthologous gene (OG) were estimated with the Codeml module of the PAML v4.10.0 package using the following settings (model = 0 and codon frequency = 2) (Yang, 2007).

Functional annotations of uncultured *Frankia* and *Protofrankia*, along with their closely related cultured counterparts, were carried out utilizing the Clusters of Orthologous Genes (COG) database (Galperin et al., 2019). The completeness of metabolic pathways was assessed using the Kyoto Encyclopedia of Genes and Genomes (KEGG) database (Kanehisa et al., 2002; Touchon and Roche, 2007). The KEGG metabolic pathways were examined through BlastKOALA (Kanehisa et al., 2016), aligning against the prokaryotes database to elucidate pathway functionalities and completeness.

Cell Wall and Capsule, Dormancy and Sporulation, and Regulation and Cell signaling, were obtained using SEED server for Rapid Annotation Subsystem Technology server (RAST) (Overbeek et al., 2014). The heatmaps were created with ComplexHeatMap package (Gu, 2022) on RStudio.

### Ecological traits extraction

Genomes of uncultured and cultured strains were submitted to the PhenDB web server,^1^ using recommended default settings with a balanced accuracy cutoff of 0.75 and a prediction confidence cutoff of 0.6 (Feldbauer et al., 2015) were employed to predict obligate or facultative intracellular lifestyles. GapMind was used to detect gene pathways for essential carbon sources (Price et al., 2021) and amino acid biosynthesis (Price et al., 2020). Functional traits extraction was mainly performed using MicroTrait, an R package streamlining the extraction of fitness traits from microbial genome sequences (Karaoz and Brodie, 2022). This tool employs profile hidden Markov models (profile-HMM) and logical operations to predict and map the protein family content within genome sequences to fitness traits. The microTrait framework depends on meticulously curated HMMs, known as microtrait-hmms, which capture the sequence diversity of protein families sourced from IMG/M. These HMMs undergo benchmarking using KEGG orthologs for trusted cutoff (TC) scores. Software dependencies include HMMER (Potter et al., 2018) and Prodigal (Hyatt et al., 2010), with additional features utilizing Infernal (Nawrocki and Eddy, 2013), tRNAscan-SE (Schattner et al., 2005) and bedtools (Quinlan, 2014). Essential data dependencies encompass microtrait-hmm (gene level profile-HMM database) and dbCAN (Huang et al., 2018)-HMMdb (domain level profile-HMM database for Carbohydrate-active enzymes). Additionally, secondary metabolite clusters were determined using AntiSMASH 7.0 (Blin et al., 2023).

## Results and discussion

Cultivating microsymbionts in axenic conditions has historically posed challenges due to specific growth requirements, nutrient dependencies, and slow growth rates (Alain and Querellou, 2009; Stewart, 2012; Pham and Kim, 2016). This study thoroughly examines previously labeled uncultured *Frankia* strains, representing a significant proportion of *Protofrankia* strains and sporulating *Frankia* microsymbionts (Sp + types) (Persson et al., 2011, 2015; Nguyen et al., 2016, 2019; Normand et al., 2017; Herrera-Belaroussi et al., 2020; Pozzi et al., 2020; Berckx et al., 2022), to determine if they exhibit barriers to isolation in axenic conditions.

### Phylogeny and taxonomy of uncultured *Frankia*

**Figure 1:**
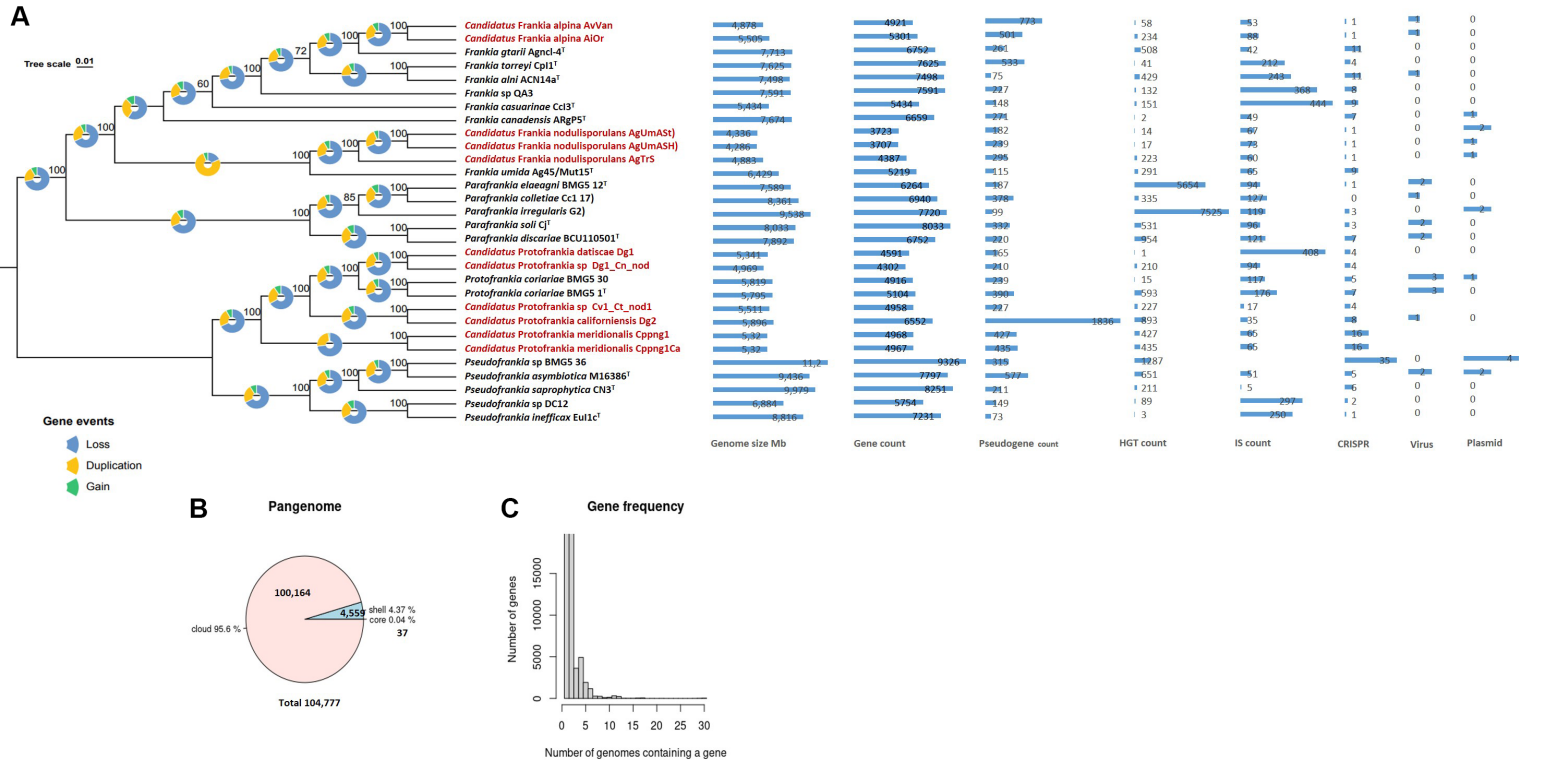
Maximum Likelihood (ML) Phylogenetic treeing based on tandem comparison of 560 single-copy genes. Gene events, including gain, loss, and duplication, were quantified using Dollo parsimony algorithms implemented in the COUNT software and presented as pie charts (Csurös, 2010). The charts depict signatures of genome evolutionary processes, encompassing size and counts for genes, pseudogenes, insertion sequences, horizontally transferred genes, plasmids, viruses, and CRISPR elements **(A)**. A pie chart representation the proportion of core and accessory genes present in the genomes **(B)**. Gene frequency displayed as the number of occurrences relative to the number of genomes containing a particular gene **(C)**. Uncultivable strains are indicated in red.

The 560 single-copy gene phylogenomic trees (Figure 1A) revealed a robust phylogenetic radiation within the *Frankiaceae* family supported by robust bootstraps and posterior probability values. This analysis clearly delineated the four genera—*Pseudofrankia*, *Protofrankia*, *Parafrankia*, and *Frankia* (Gtari, 2022). Mash-based analyses, utilizing MinHash sketches and Jaccard indices for shared k-mers, enabled cohesive clustering of the 30 genomes (Supplementary Figure S1), ensuring a robust alignment of type strain species classifications within their respective genera. Significantly, the most elevated similarities observed for *Frankia umida* Ag45_Mut15^T^ and *Candidatus* Frankia nodulisporulans members were 97.15% with AgTrS, 96.80% with AgUmASH1, and 96.82% with AgUmASt1 (Supplementary Figure S1). Digital DNA:DNA hybridization (dDDH) between *Candidatus* Frankia nodulisporulans members and Ag45_Mut15^T^ yielded 73.7% ([70.7–76.5]), designating AgTrS, AgUmASH1, and AgUmASt1 as members of *Frankia umida* species (Chun et al., 2018). *Candidatus* Protofrankia datiscae Dg1 and *Protofrankia* symbiont of *Coriaria nepalensis* Dg1_Cn_nod exhibited genomic similarities of 96.72 and 96.65%, respectively, with *Protofrankia coriariae* BMG5.1^T^ (Supplementary Figure S2). dDDH values ranged from 66.5% ([63.5–69.3]) to 67.5% ([64.5–70.3]), classifying them as a borderline taxon. *Candidatus* Frankia alpina strains AiOr and AvVan displayed values of 95.58 and 95.86%, respectively, with *Frankia gtarii* strain Agncl-4^T^. However, dDDH values of 56.90% ([54.1–59.6%]) for AiOr and 4.50% ([51.8–57.2%]) for AvVan suggest genomic distinctions.

This observation aligns with findings in microbial ecology, where the uncultivability of certain taxa is not solely attributed to evolutionary divergence but may involve a complex interplay of ecological adaptations and genomic features (Stewart, 2012). It challenges the conventional notion that uncultured microbes represent entirely novel lineages, emphasizing the need for a nuanced understanding of microbial diversity and ecology (Connon and Giovannoni, 2002). The potential genomic continuity hints at shared ancestry, possibly driven by environmental factors influencing microbial evolution (Shade et al., 2014).

### Genome structure and evolution of uncultured *Frankia*

**Figure 2:**
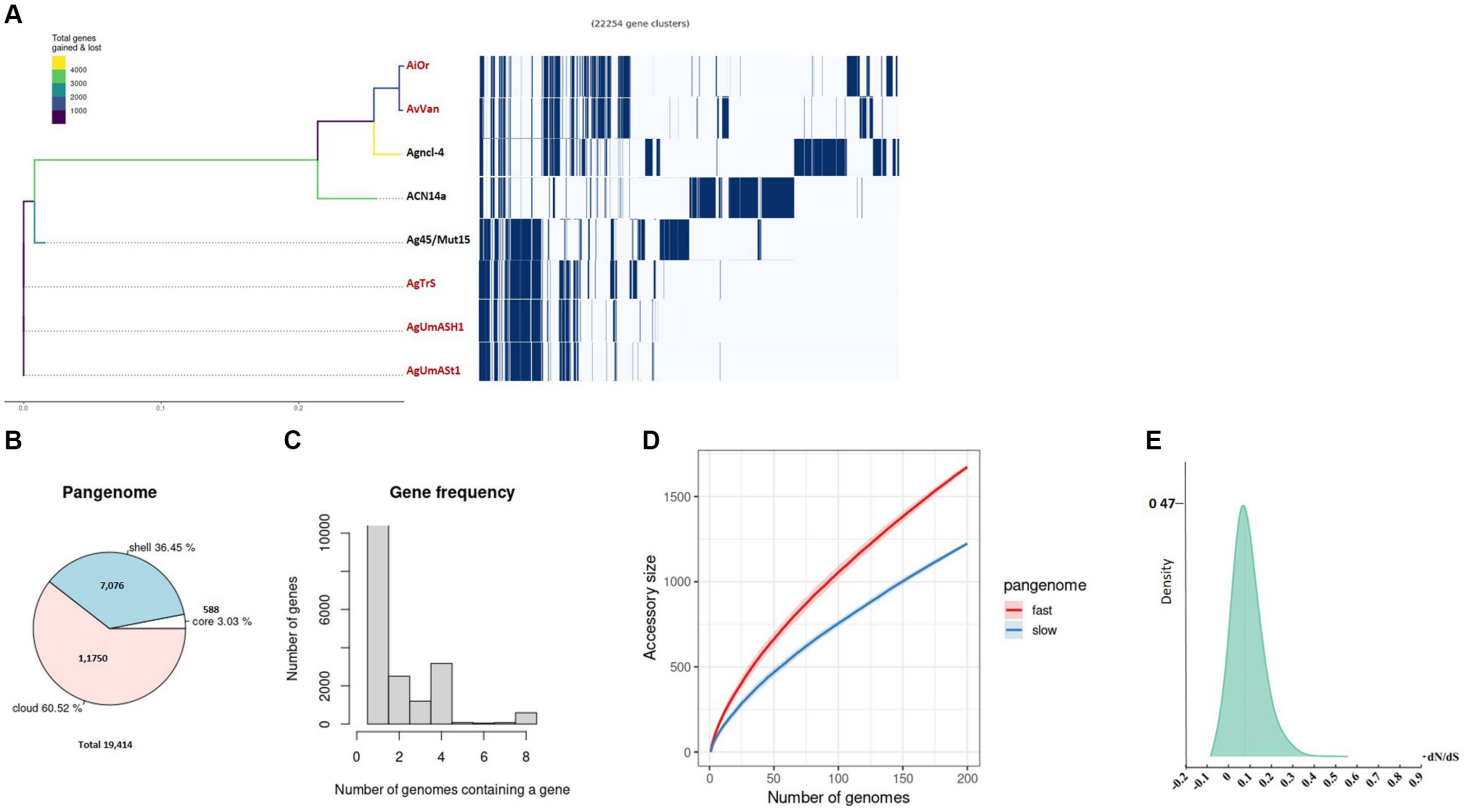
Pangenome analysis was conducted on uncultured *Frankia* strains and their closely related cultured counterparts. **(A)** The phylogenetic branching of uncultured *Frankia* is depicted, accompanied by the gene presence/absence matrix resulting from pangenome analysis, where each row represents the gene profile of an individual genome. A pie chart illustrates the proportion of core and accessory genes across the analyzed genomes **(B)**. Gene frequency is presented as the number of occurrences relative to the number of genomes containing a particular gene **(C)**. Pangenome accumulation curves for accessory genes, showcasing both fast and slow accumulation dynamics, are shown **(D)**. Density plot of dN/dS values **(E)**. Uncultivable strains are indicated in red.

**Figure 3:**
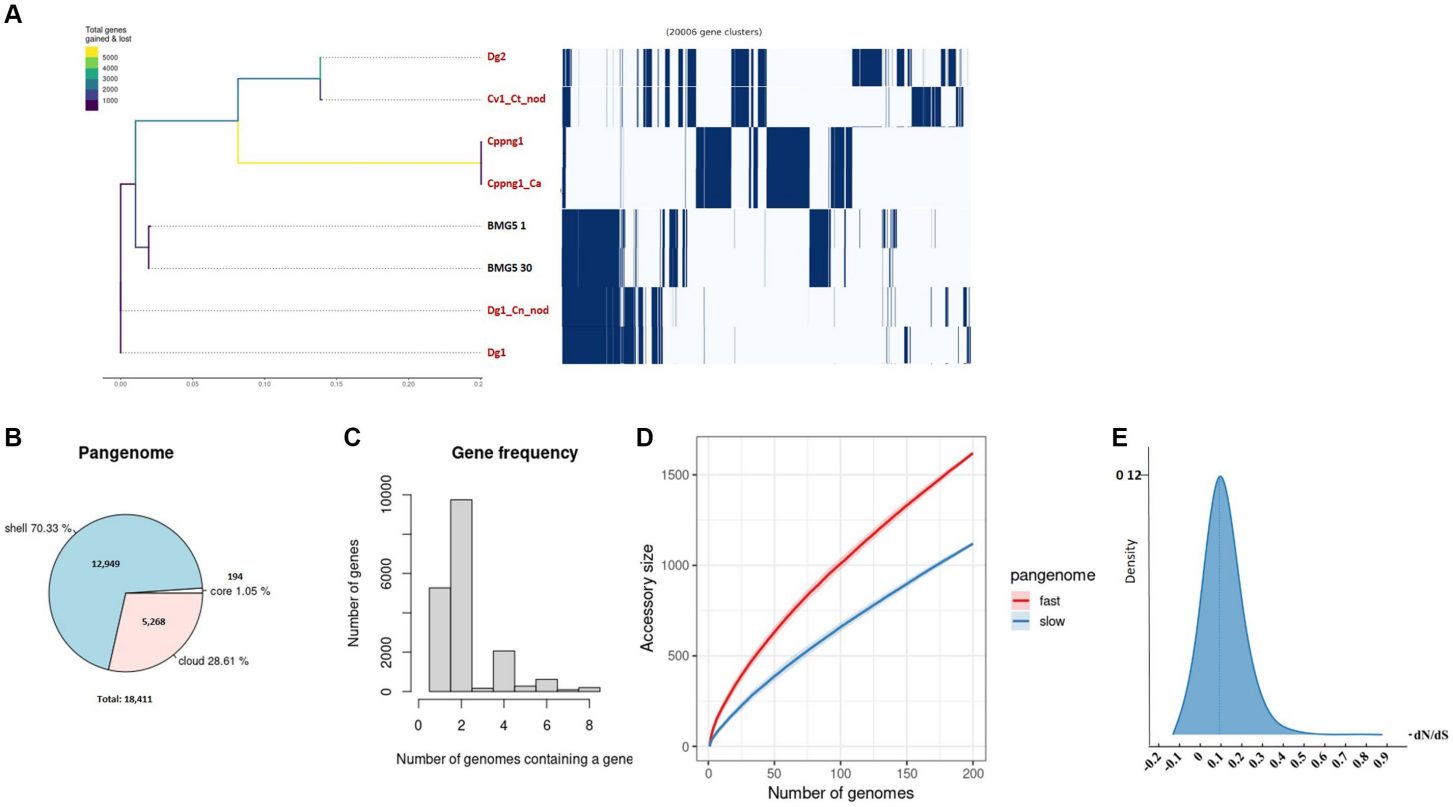
An exploration of the pangenome involved the examination of uncultured *Protofrankia* strains and their closely related cultured counterparts. The phylogenetic branching of uncultured *Protofrankia* is portrayed, accompanied by a gene presence/absence matrix resulting from the pangenome analysis, where each row delineates the gene profile of an individual genome **(A)**. Additionally, a pie chart visually represents the proportion of core and accessory genes distributed across the analyzed genomes **(B)**. Gene frequency as the number of occurrences relative to the number of genomes containing a specific gene **(C)**. The dynamics of pangenome accumulation for accessory genes, displaying both rapid and gradual accumulation **(D)**. Density plot of dN/dS values **(E)**. Uncultivable strains are indicated in red.

As stated previously, an obligate intracellular lifestyle may lead to genome reduction, making microorganisms uncultivable in the lab (Casadevall, 2008; Rodríguez-Gijón et al., 2022). Genomic comparisons between cultured and uncultured *Frankia* strains revealed consistent genome size differences (0.924–3.427 Mb) (Figure 1A), a trend also observed among cultivated strains associated with host plant speciation (Normand et al., 2007; Tisa et al., 2016). Insertion sequences, horizontal gene transfer, viral interactions, plasmid dynamics, and CRISPR systems collectively drive genome evolution (Casadevall, 2008; Sloan and Moran, 2013; Brito, 2021; Arnold et al., 2022). These elements play a role in rearranging genomes, introducing novel genetic material, and the CRISPR systems serve as a defense mechanism against integration (Nussenzweig and Marraffini, 2020). Diverse adaptive strategies were evident in gene density (ratio of the number of genes per number of base pairs) variations, with some uncultured *Frankia *strains exhibiting high gene densities (Figure 1A), possibly indicating specialized adaptations (Nakabachi et al., 2006; Kuo et al., 2009; Kuo and Ochman, 2010). Pseudogenes identified in the entire set of 30 genomes predominantly displayed features such as incompleteness, frameshifts, multiple issues, internal stop codons, with fewer instances of ambiguous residues (Supplementary Table S1). The heightened presence of pseudogenes in Dg2 implies continuous evolutionary dynamics, likely influenced by specific environmental pressures. Furthermore, the KEGG distribution of pseudogenes primarily associated them with “carbohydrate metabolism,” followed by “Genetic information processing,” and “Unclassified genetic information processing” (Supplementary Figure S3). The examination of gene events in the 30 analyzed *Frankiaceae* genomes unveiled a predominant sequence of occurrences, primarily involving loss, followed by duplication, and subsequently, gain (Figure 1A). Notably, in the case of uncultured *Frankia*, the total number of gene gains and losses was comparatively lower than in their cultured counterparts (Figure 2A). Specifically, the counts range from 1,000 to 2,000 for uncultured *Frankia*, whereas they range from 3,000 to 4,000 for cultured strains. Conversely, in the *Protofrankia* group, cultured strains exhibit similar or lower counts (1,000–2,000) compared to their uncultured counterparts (1,000–5,000) (Figure 3A).

Gene-gain events are contingent on the rates of horizontal gene transfer and gene duplication, while gene-loss events hinge on the rates of gene inactivation and deletion (Bordenstein and Reznikoff, 2005). The occurrence of various horizontal gene transfers events, regardless of the cultivation status, underscores the inherent ability of *Frankia* strains, whether cultured or uncultured, to facilitate the exchange of genetic material. This phenomenon is intricately linked to the symbionts’ ability to establish contact not only with conspecific strains but also with other bacterial species, a possibility that extends even within the confines of actinorhizal nodules (Ghodhbane-Gtari et al., 2021).

### Pangenome analysis

The pangenome categorizes genes into three groups: core genes, shared universally across all strains (subdivided into core and soft core genes); accessory genes, found in subsets of strains (subdivided into shell and cloud genes); and unique/singleton genes, exclusive to individual strains (Tettelin et al., 2005). Core genes are essential for basic functions (Tettelin et al., 2005; Kim et al., 2020), while accessory and unique genes reflect adaptations to environments, hosts, or lifestyles (Zhang and Sievert, 2014; Nelson and Stegen, 2015), which may influence their propensity for growth under axenic conditions.

Our investigation of 30 *Frankiaceae* genomes revealed a diverse genomic landscape with 37 universally present core genes, 17 soft core genes in a substantial subset, and 4,559 shell genes (Figure 1B). Additionally, 100,164 cloud genes indicate a dynamic genetic reservoir within this taxonomic group, forming a pangenome of 104,777 genes that offers insights into genetic diversity, adaptability, and evolutionary dynamics.

Analysis of *Frankia* and *Protofrankia* revealed distinct genetic landscapes. The pangenome analysis of *Frankia* strains revealed a total of 19,414 genes (Figure 2B). Among these, 588 core genes were present in 99–100% of the strains, indicating their essential role. The pangenome includes 7,076 shell genes (15–95% presence), contributing to functional diversity, and 11,750 cloud genes (less than 15% presence), highlighting extensive genetic variability.

The pangenome analysis of *Protofrankia* strains revealed a total of 18,411 genes (Figure 3B). Among these, 194 core genes were present in 99–100% of the strains, indicating their essential role. The pangenome includes 12,949 shell genes (15–95% presence), contributing to functional diversity, and 5,268 cloud genes (less than 15% presence), highlighting extensive genetic variability.

No soft-core genes (95–99% presence) were found in the analyzed *Frankia* and *Protofrankia* strains. Soft-core genes are particularly relevant in analyses involving draft genomes as some genes may be missing in draft genomes due to their lower assembly quality than completely assembled genomes (Nelson and Stegen, 2015). The difference in core genes (588 vs. 194) between *Frankia* and *Protofrankia* can be attributed to several factors. Sampling bias is a significant factor, as core gene numbers vary with the diversity and quantity of strains analyzed. *Protofrankia* likely shows a smaller core genome due to fewer sequenced strains and closer genetic relationships compared to *Frankia* (Nouioui et al., 2014). Methodological differences also play a crucial role, with core genome estimates significantly impacted by genome incompleteness, fragmentation, and contamination, which are more prevalent in metagenome-assembled genomes (MAGs) compared to single isolate assembled genomes (Li and Yin, 2022). Additionally, genomic diversity affects core genome size and composition; *Frankia* species likely possess a larger core genome due to greater genomic diversification across varied environments, unlike *Protofrankia* species (Gtari, 2022).

Analyses of all three sets—*Frankia*, *Protofrankia*, and *Frankiaceae*—have revealed open pangenomes (Figures 1C, 2C,D, 3C,D), as indicated by the continuous discovery of new gene families with the addition of new genomes (Costa et al., 2020). The configuration of a pangenome, whether open or closed, is intricately tied to the lifestyle of bacterial species, with sympatric species thriving in interactive, communal environments demonstrating open pangenomes characterized by a continuous influx of new genes through horizontal gene transfer (Medini et al., 2005). Conversely, allopatric species, residing in isolated settings, typically exhibit smaller, closed pangenomes (Medini et al., 2005; Georgiades and Raoult, 2011).

Three distinct types of evolutionary selection processes, based on codon substitution, include positive selection (dN/dS > 1), purifying selection (dN/dS < 1), and neutral selection (dN/dS = 1) (Spielman and Wilke, 2015). The average dN/dS ratios for genes in both *Frankia* and *Protofrankia*, whether cultured or uncultured, were consistently found to be less than 1 (Figures 2E, 3E). This pattern suggests that all analyzed genes are subject to purifying selection, signifying the preservation of functional integrity and evolutionary stability across the analyzed strains (Sarkar and Sen, 2022).

### Homologous genes present uniquely in cultured *Frankia*

The absence of specific genes in uncultivated microsymbionts, as revealed by Orthovenn3 analysis, highlights potential factors contributing to the challenges associated with their cultivability. In cultivated *Frankia* strains, 521 unique genes, with functions spanning transcription regulation, fatty acid biosynthesis, and defense responses, suggest enhanced capabilities in cellular regulation, metabolic activities, and defense mechanisms (Tables 2, 3; Supplementary Figure S4). The molecular functions of these genes, including oxidoreductase and ATPase activities, underscore their vital roles in cellular processes. Similarly, *Protofrankia* exhibits 825 exclusive genes, emphasizing its distinct genetic repertoire (Table 3; Supplementary Figure S5). These genes were predominantly associated with transcription regulation and transmembrane transport, indicating potential adaptations to environmental cues and nutrient uptake strategies. The inclusion of functions related to sporulation, phospholipid transport, oxidoreductase, ATPase activities, DNA binding, and metal ion binding in *Protofrankia’s* exclusive gene set suggests a diverse array of cellular processes. The absence of these genes in uncultivated strains may signify a less ability compared to cultivated strains to regulate gene expression, perform essential metabolic functions, and adapt to diverse environmental conditions, all of which are crucial for successful laboratory cultivation.

**Table 2:** GO enrichment of proteins present in cultured and absent in uncultured *Frankia* strains.

GO term	Protein count	Description	Ontology	*p*-values
GO:0006355	17	Regulation of transcription, DNA-templated	biological_process	6.663500585975715 e-64
GO:0006633	11	Fatty acid biosynthetic process	biological_process	7.95951272204721 e-09
GO:0017000	10	Antibiotic biosynthetic process	biological_process	7.927208309074664 e-17
GO:0051607	8	Defense response to virus	biological_process	0.0002428283280399067
GO:0006099	6	Tricarboxylic acid cycle	biological_process	2.2038068560022853 e-12
GO:0030435	6	Sporulation resulting in formation of a cellular spore	biological_process	8.642292890190224 e-08
GO:0046677	4	Response to antibiotic	biological_process	5.3922645948956175 e-31
GO:0009405	3	Pathogenesis	biological_process	6.955094934645369 e-17
GO:0044873	3	Lipoprotein localization to membrane	biological_process	0.008590721131043375
GO:0009164	3	Nucleoside catabolic process	biological_process	0.008590721131043375
GO:0019439	2	Aromatic compound catabolic process	biological_process	3.6751977821591035 e-08
GO:0006631	2	Fatty acid metabolic process	biological_process	5.746754744693072 e-07
GO:0006352	3	DNA-templated transcription, initiation	biological_process	2.8772858457099777 e-06
GO:0016114	2	Terpenoid biosynthetic process	biological_process	5.3263026401023245 e-06
GO:0055114	2	Oxidation–reduction process	biological_process	3.014345799313272 e-05
GO:0030497	2	Fatty acid elongation	biological_process	4.615037074227507 e-05
GO:0045232	2	S-layer organization	biological_process	0.016733170871222863
GO:0005886	6	Plasma membrane	cellular_component	9.023797064238693 e-26
GO:0016491	11	Oxidoreductase activity	molecular_function	7.735224554095876 e-42
GO:0046872	9	Metal ion binding	molecular_function	1.2421362299588434 e-29
GO:0016887	5	ATPase activity	molecular_function	7.142362787033378 e-14
GO:0043565	4	Sequence-specific DNA binding	molecular_function	0.00493561331449674
GO:0008061	3	Chitin binding	molecular_function	0.008590721131043375
GO:0003700	2	Sequence-specific DNA binding transcription factor activity	molecular_function	1.9570856204302962 e-10

**Table 3:** GO enrichment of proteins present in cultured and absent in uncultured *Protofrankia* strains.

GO term	Protein count	Description	Ontology	*p*-values
GO:0006355	6	Regulation of transcription, DNA-templated	biological_process	5.062598895569772 e-37
GO:0055085	5	Transmembrane transport	biological_process	1.0277209374700805 e-26
GO:0030435	4	Sporulation resulting in formation of a cellular spore	biological_process	5.046482433806986 e-07
GO:0015914	4	Phospholipid transport	biological_process	0.0005561264004201647
GO:0006633	4	Fatty acid biosynthetic process	biological_process	1.089172842661041 e-11
GO:0046677	2	Response to antibiotic	biological_process	1.1207957183541802 e-21
GO:0006260	2	DNA replication	biological_process	1.7912991284821486 e-07
GO:0006777	2	Mo-molybdopterin cofactor biosynthetic process	biological_process	0.008059777854697709
GO:0006281	2	DNA repair	biological_process	2.3291849989590412 e-05
GO:0006099	2	Tricarboxylic acid cycle	biological_process	4.709376529576225 e-10
GO:0006313	2	Transposition, DNA-mediated	biological_process	2.163482211983071 e-08
GO:0055114	2	Oxidation–reduction process	biological_process	0.00013337251121081238
GO:0005886	2	Plasma membrane	cellular_component	2.3387610709332023 e-15
GO:0016491	10	Oxidoreductase activity	molecular_function	1.532736552223947 e-16
GO:0016705	6	Oxidoreductase activity, acting on paired donors, with incorporation or reduction of molecular oxygen	molecular_function	1.750694992145492 e-10
GO:0016887	6	ATPase activity	molecular_function	3.598716601114218 e-08
GO:0003677	4	DNA binding	molecular_function	4.2368584396231013 e-16
GO:0046872	4	Metal ion binding	molecular_function	7.974237522227637 e-27
GO:0004674	2	Protein serine/threonine kinase activity	molecular_function	5.086789909828374 e-14
GO:0043565	2	Sequence-specific DNA binding	molecular_function	0.00024753581859025836
GO:0016627	2	Oxidoreductase activity, acting on the CH-CH group of donors	molecular_function	0.000391191404846266
GO:0008061	2	Chitin binding	molecular_function	0.023850892434054655
GO:0004090	2	Carbonyl reductase (NADPH) activity	molecular_function	0.023850892434054655

**Table 4:** Main predicted features of uncultured *Frankia* and *Protofrankia* and their closely related counterparts using phendb server.

Strain	Gram stain	Aerobe	sporulation	Symbiont	Saccharolytic	Acetic acid production	Ethanol production	motility	*nif*
Dg1	+	+	+	−	+	+	+	−	+
BMG5.1	+	+	+	−	+	+	+	−	+
BMG5.30	+	+	+	−	+	+	+	−	+
Cv_Ct_nod1	+	+	+	−	+	+	+	−	+
Dg2	+	+	+	−	+	+	+	−	+
Cm1_Cm_nod	+	+	+	−	+	+	+	−	+
Cppng1_Ca_nod	+	+	n.d.	−	+	+	+	−	+
Cppng1	+	+	n.d.	−	+	+	+	−	+
ACN14	+	+	+	−	+	n.d.	+	−	+
Ag45/Mut15	+	+	+	−	+	+	+	−	+
Agncl-4	+	+	+	−	+	+	+	−	+
AgUmASt1	+	+	n.d.	−	+	+	+	−	+
AgTrS	+	+	n.d.	−	+	+	+	−	+
AgUm	+	+	n.d.	−	+	+	+	−	+
AvVan	+	+	n.d.	−	+	+	+	−	+
AiOr	+	+	n.d.	−	+	+	+	−	+

Default settings parameters were used with a balanced accuracy cutoff of 0.75 and a prediction confidence cutoff of 0.6 (Feldbauer et al., 2015).

n.d., not determined.

## Vital cellular components and central metabolic pathways

### Cell wall and capsule

Bacteria with unconventional cell wall structures can pose challenges for cultivation (Van Teeseling et al., 2015; Markova, 2020; Megrian et al., 2022). Apart from the “Exoenzymes regulatory protein AepA in lipid-linked oligosaccharide synthesis cluster,” absent in certain uncultured *Frankia* strains (AiOr, AvVan, AgTrS, and AgUmASH1), and the “Apolipoprotein N-acyltransferase (EC 2.3.1.-) in lipid-linked oligosaccharide synthesis cluster,” lacking in strain AiOr, all components of the “Cell wall and Capsule pathway” were detected in uncultured strains of *Frankia* and *Protofrankia* compared to their cultivated counterparts (Supplementary Figures S6, S7). This finding suggests that the uncultured *Frankia* and *Protofrankia* strains are capable of generating intact Cell Wall and Capsule structures.

### Dormancy and sporulation

Dynamics of dormancy and sporulation, contributing to our understanding of the cultivability of specific strains (Kell and Young, 2000; Wörmer et al., 2019). Except for the “Spore pigment biosynthetic cluster in Actinomycetes. Polyketide hydroxylase WhiE VIII,” absent in strain Dg2, all components of the “Dormancy and Sporulation, no subcategory” are detectable in uncultured strains of *Frankia* and *Protofrankia* compared to their cultured counterparts. This indicates functional integrity within this pathway across the uncultured strains (Supplementary Figures S6, S7).

### Regulation and signaling

Dearth of regulatory elements in obligate lifestyle stems from the loss of structural genes during reductive evolution is a consequence of adapting to narrow ecological niches (Wilcox et al., 2003). Regulation and Cell Signaling, both cultured and uncultured *Frankia* strains share cAMP signaling components and exhibit consistent expression of essential elements (Supplementary Figure S6). The cAMP signaling, may indicate common regulatory features, and disruptions in these components can influence cultivation success (Vartoukian et al., 2010). The Stringent Response, Programmed Cell Death, and Toxin-Antitoxin Systems are prevalent in all analyzed genomes which contribute to bacterial behavior (Barer and Harwood, 1999; Prozorov and Danilenko, 2010). Uncultured *Protofrankia*, and *Frankia* strains along with cultured related strains share all “Regulatory and cell Signaling” features (Supplementary Figure S7). Common enzymatic activities in the cAMP pathway and consistent presence of regulatory proteins underscore similarities in regulatory mechanisms across these strains. Shared functionalities in the stringent response, (p)ppGpp metabolism, play a crucial role in bacterial survival under varying conditions (Fagen et al., 2014), and sporulation-related proteins further highlight commonalities in these pathways (Galperin et al., 2012; Browne et al., 2016).

### Central metabolic pathways

The absence or deregulation of specific central metabolic pathways is known to hinder the cultivability of certain bacterial taxa (Pande and Kost, 2017; Bodor et al., 2020; Zhao et al., 2023). Analyzing metabolic pathway data from uncultured *Frankia* and *Protofrankia* strains alongside their closely related cultured counterparts reveals that crucial central metabolic pathways are largely intact across these organisms (Supplementary Figure S8). Key pathways such as glycolysis (Embden-Meyerhof pathway), gluconeogenesis, the citrate cycle (TCA cycle), and the pentose phosphate pathway exhibit high completeness levels. Furthermore, pathways involved in pyruvate oxidation, fatty acid biosynthesis, and various amino acid biosynthesis pathways are also well-represented with substantial completeness (Supplementary Figure S9).

These findings indicate a diverse metabolic repertoire uncultured *Frankia* and *Protofrankia* compered to their cultured counterparts, enabling efficient utilization of diverse carbon sources and synthesis of essential biomolecules critical for growth and survival. The presence of these pathways underscores the metabolic flexibility of these bacterial strains, likely facilitating their adaptation to different environmental conditions and ecological niches. However, notable exceptions are observed in the uncultured *Protofrankia* Dg2 strain, particularly in pathways such as the Entner-Doudoroff pathway and certain aspects of nucleotide sugar biosynthesis, which display partial completeness. These observations suggest specific metabolic adaptations or limitations in these pathways within the *Protofrankia* Dg2 strain.

## Predictive ecological traits

**Figure 4:**
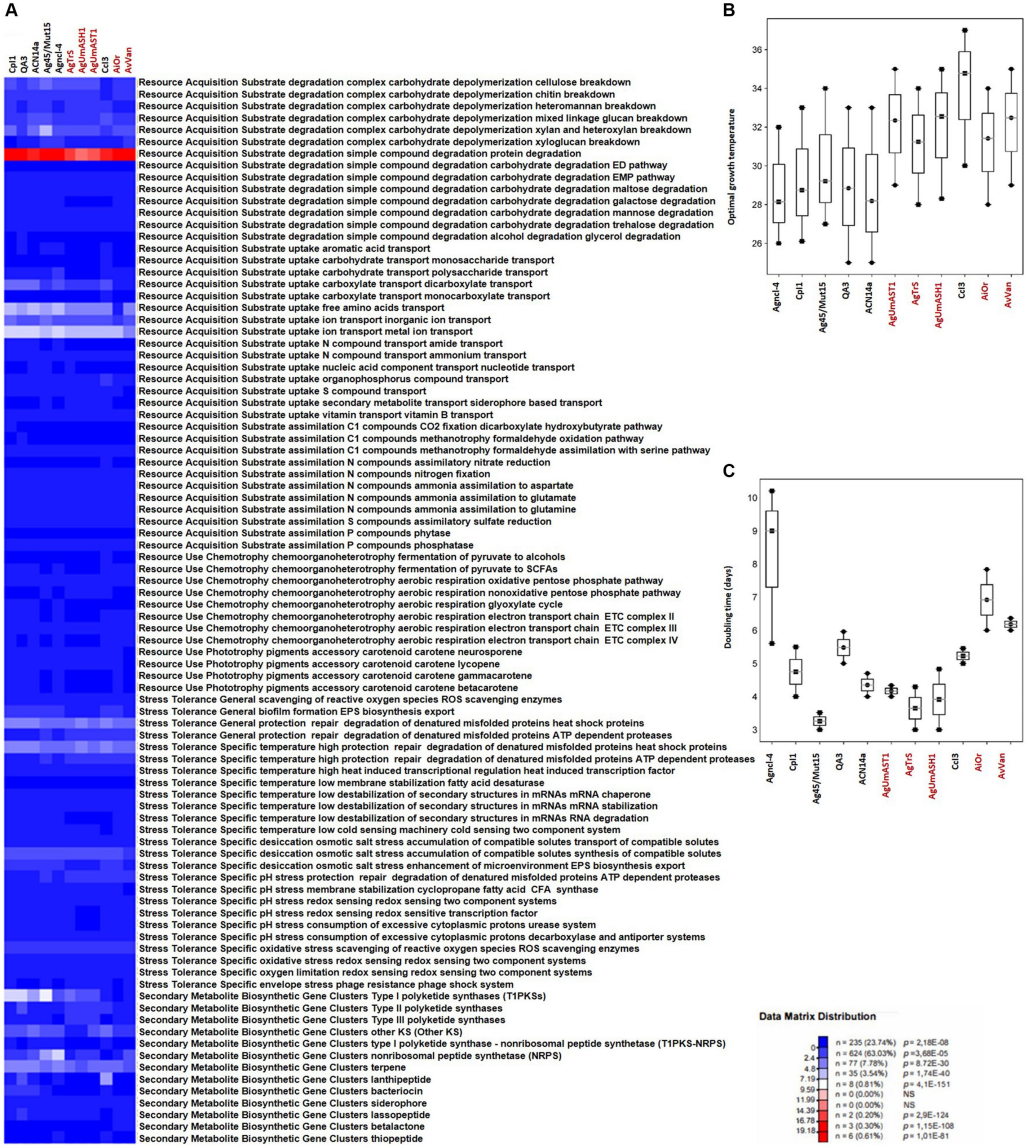
Overview of microbial traits generated by the MicroTrait pipeline, which extracts fitness traits from microbial genome sequences (Karaoz and Brodie, 2022). This figure shows ecological trait profiles for specific guilds in uncultured *Frankia* (AgUmAST1, AgTrS, AgUmASH1, AiOr, and AvVan) and their cultured counterparts, covering resource acquisition, utilization, and stress tolerance. In microTrait, each protein family is a Boolean variable (1 if detected, 0 if not), determined by microTrait-HMMs (Supplementary Table S4), with traits defined by rules using these variables. Secondary metabolite clusters identified using AntiSMASH 7.0 enhance trait visualization **(A)**. Predictions for optimal temperature **(B)** are based on genomic features (Sauer and Wang, 2019), and doubling time **(C)** is predicted using codon-usage bias in ribosomal protein genes (Vieira-Silva and Rocha, 2010; Weissman et al., 2021). The top panel shows the statistical significance of comparing mean trait values across guilds.

**Figure 5:**
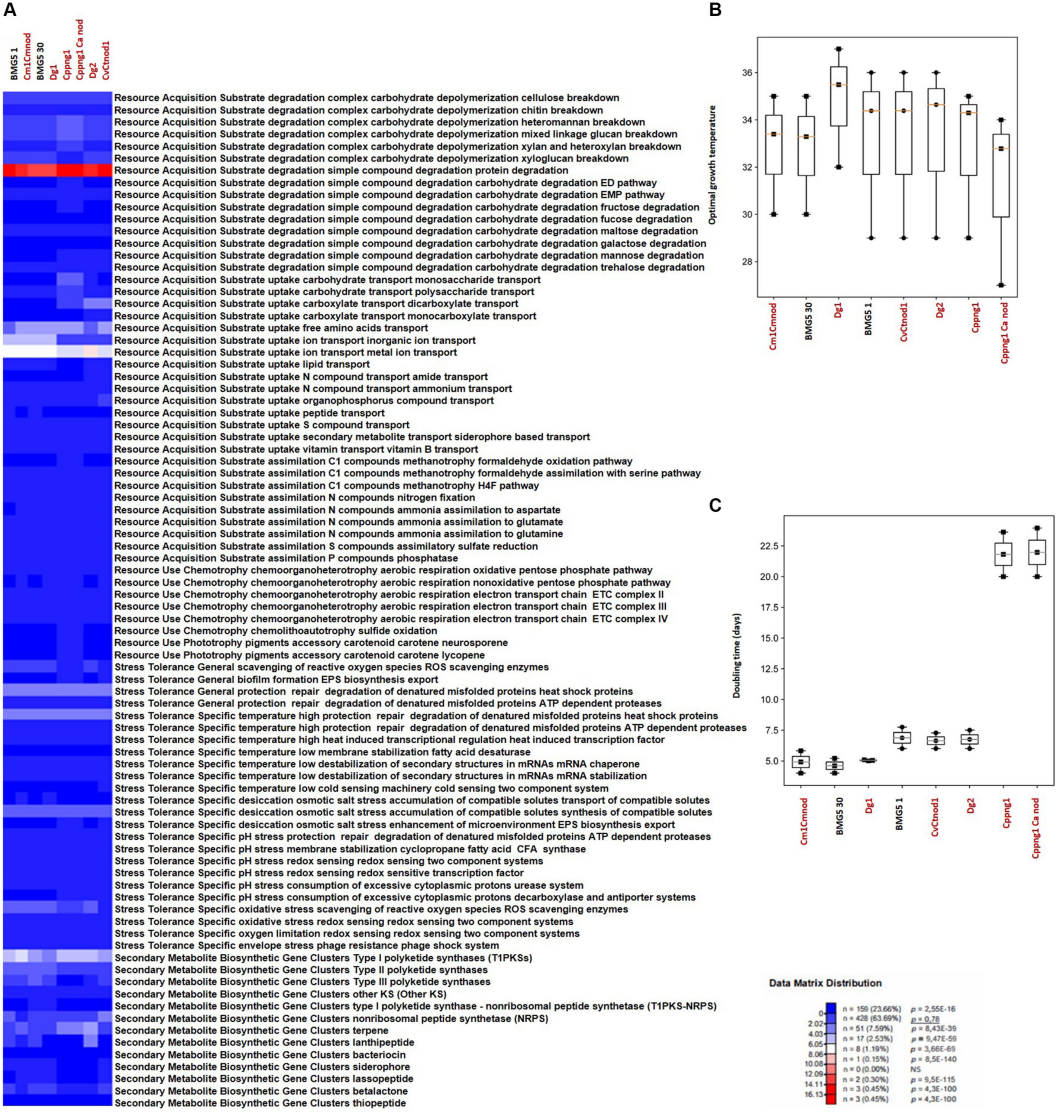
Overview of microbial traits generated by the MicroTrait pipeline, which extracts fitness traits from microbial genome sequences (Karaoz and Brodie, 2022). This figure showcases ecological trait profiles for specific guilds in uncultured *Protofrankia* (Cm1Cmnod, Dg1, CvCtnod1, Dg2, Cppng1, and Cppng1 Ca nod) and their cultured counterparts, covering resource acquisition, utilization, and stress tolerance. In microTrait, each protein family is a Boolean variable (1 if detected, 0 if not), determined by microTrait-HMMs (Supplementary Table S5), with traits defined by rules using these variables. Secondary metabolite clusters identified using AntiSMASH 7.0 enhance trait visualization **(A)**. Predictions for optimal temperature (Sauer and Wang, 2019) **(B)** and doubling time (Vieira-Silva and Rocha, 2010; Weissman et al., 2021) **(C)** provide comprehensive insights. The top panel shows the statistical significance of comparing mean trait values across guilds.

Conventional genomic analyses often fall short in capturing the ecological intricacies of uncultured microorganisms. The current trend emphasizes organization into a metabolomic blueprint that encapsulates ecological traits (Giovannoni et al., 2014; Lewis et al., 2021; Gtari et al., 2024). This approach, facilitated by advanced bioinformatics tools and algorithms, enables the unveiling of metabolic profiles and the interpretation of cultivability/uncultivability of a given strain. Here we used MicroTrait, an R package (Karaoz and Brodie, 2022) to generate trait profiles for the defined guilds (guilds × traits), with mean (Figures 4A, 5A). Ecological guilds denote groups of species or strains with similar resource-use strategies. AntiSMASH 7.0 (Blin et al., 2023), GapMind (Price et al., 2020, 2021) and PhenDB (Feldbauer et al., 2015), to extract other fitness traits from *Frankia* genome sequences.

### Resource acquisition traits

In contrast to obligate symbionts and pathogens that rely on hosts for nutrients, saprophytic and facultative symbiotic microorganisms can utilize decaying organic matter (Ray et al., 2009; Salje, 2021). Their efficient decomposition involves rich enzymatic sets for breakdown, membrane transporters, and uptake through central metabolic pathways, providing nutrients and energy for cellular functions (Cao et al., 2023). Comparing cultured and uncultured strains in complex carbohydrate depolymerization and resource acquisition pathways reveals shared features correlating with similar nutritional requirements (Figures 4A, 5A). Both groups exhibit proficiency in breaking down complex carbohydrates (cellulose, chitin, heteromannan, mixed linkage glucan, xylan, and xyloglucan) and degrading simple compounds (proteins, EMP pathway carbohydrates, maltose, galactose, mannose, trehalose, and glycerol). For substrate uptake, both groups show similarities in transporting aromatic acids, monosaccharides, polysaccharides, dicarboxylates, monocarboxylates, free amino acids, inorganic ions, metal ions, amides, ammonium, nucleotides, organophosphorus compounds, sulfur compounds, siderophores, and vitamin B. Both groups display balanced assimilation of C1 compounds via CO2 fixation through the dicarboxylate hydroxybutyrate pathway, methanotrophy utilizing formaldehyde oxidation or assimilation with serine, assimilatory nitrate reduction, nitrogen fixation, ammonia assimilation to aspartate, glutamate, or glutamine, assimilatory sulfate reduction, and phosphatase-mediated phosphorus compound assimilation. They also exhibit the ability to ferment pyruvate to short-chain fatty acids (SCFAs) and use the oxidative pentose phosphate pathway for aerobic respiration, sharing the presence of the electron transport chain (ETC) complex III. In terms of phototrophy, both cultured and uncultured strains produce the accessory carotenoid pigments neurosporene and lycopene. However, specific pathways like the aerobic respiration nonoxidative pentose phosphate pathway and the glyoxylate cycle are found in various cultured strains but are absent in some uncultured strains. Additionally, the electron transport chain ETC complex IV is present in cultured but missing in all uncultured strains (Figures 4A, 5A). These distinctions highlight the enhanced metabolic diversity and adaptability of cultured strains under controlled conditions.

### Stress tolerance

While host tissues can impose chemical and/or physical stresses, notably through the host immune system’s response to microbe-associated molecular patterns (Schwartzman and Ruby, 2016; Bénard et al., 2020), an obligate lifestyle can be regarded as an adaptive response to narrow ecological niches, providing a buffered environment conducive to maintaining homeostasis. This adaptation involves the regulation of pH, temperature, and osmotic stress, enabling the host to efficiently mitigate these environmental challenges.

The comparison of stress tolerance between cultured and uncultured *Frankia* strains showed extensive shared behaviors (Figure 4A). Both groups excel in scavenging reactive oxygen species (ROS) and maintaining redox balance, indicating strong defenses against oxidative stress (score 1). Cultured strains, however, exhibit enhanced biofilm formation and EPS biosynthesis/export compared to uncultured strains, potentially increasing their ability to form structured communities. Under heat stress, both groups demonstrate robust protein protection and repair mechanisms involving ATP-dependent proteases. Cultured strains also display superior cold-sensing machinery, highlighting their adaptability to temperature fluctuations. In desiccation, osmotic, and salt stress, both groups show similar proficiency in compatible solute synthesis and transport, suggesting shared strategies for osmoregulation. All strains possess redox-sensing systems for pH adaptation, crucial for survival in diverse environmental conditions. Cultured *Protofrankia* strains exhibit higher enzyme counts in ROS scavenging, suggesting distinct oxidative stress response mechanisms compared to uncultured strains (Figure 5A). While biofilm formation varies, both groups demonstrate comparable abilities in protecting, repairing, and degrading denatured proteins (score 4–5), emphasizing their resilience under stress conditions. Temperature-specific, osmotic, salt stress responses, and pH stress responses show variations among strains but consistent patterns within each group. Importantly, all strains share capabilities in ROS scavenging, redox-sensing, and response to oxygen limitation and envelope stress, including phage resistance, enhancing their resilience in challenging environments.

It is important to note that cultured *Protofrankia* strains have showcased a notable preference for alkalophilic conditions, setting them apart from other cultured members of the *Frankiaceae* family (Gtari et al., 2015). This investigation further delineates these distinctions, revealing that *Frankia* strains generally display a heightened capacity for the consumption of excessive cytoplasmic protons when compared to *Protofrankia*. Significantly, specific enzyme systems implicated in pH stress responses exhibit variations between the two genera. *Frankia* strains, in particular, tend to favor misfolded proteins ATP-dependent proteases, decarboxylase, and antiporter systems, in contrast to *Protofrankia*. These discerning findings underscore the unique adaptive strategies employed by *Frankia* and *Protofrankia* in response to pH stress, potentially elucidating the alkalophilic preference observed in *Protofrankia*.

### Secondary metabolite gene clusters

Secondary metabolites, distinct from primary metabolites, are characterized by their low molecular mass and do not play direct roles in the growth, development, or reproduction of the producing organism. However, they are often produced during the late growth phase of the microorganisms and play crucial roles in adaptation, competition, and defense mechanisms, conferring fitness advantages (O’Brien and Wright, 2011). These compounds are versatile in their biological functions, participating in inter-microbial warfare through processes such as antibiotic biosynthesis and resistance (Perry et al., 2022) and bacteriocin production (Riley and Wertz, 2002). Additionally, they can act as mediators of cross-species mutualism (O’Brien and Wright, 2011), or even participate in both processes, as seen with siderophores (Kramer et al., 2020). It’s crucial to note that obligate symbiotic bacteria, such as those residing within root nodules, may produce a distinct set of secondary metabolites compared to facultative symbtiotic and cultivable counterparts (Pinto-Carbó et al., 2016). This difference arises from their specialized lifestyle, which prioritizes mutualistic interactions within host environments rather than competitive survival in broader ecological settings. As a result, these bacteria may focus on producing metabolites that support their symbiotic relationship with the host plant, such as compounds involved in nitrogen fixation or modulation of plant physiology, rather than defensive or competitive compounds typically found in free-living bacteria (Gtari et al., 2015).

The analysis of biosynthetic gene clusters in both cultivated and uncultivated *Frankia* strains revealed that both groups exhibit the presence of polyketide synthases (t2PKS, t3PKS, otherKS), non-ribosomal peptide synthetases (NRPS), and genes related to terpene, siderophore, and lassopeptide biosynthesis, suggesting a common potential for secondary metabolite production (Figure 4A). However, cultured strains show higher counts in specific clusters, including t1PKS1, NRPS, and terpene genes, indicating potential metabolic advantages in these pathways. Conversely, the absence of specific clusters, such as lassopeptide and betalactone, in uncultivated strains suggests potential ecological adaptations and differences in secondary metabolite production. For cultivated and uncultivated *Protofrankia* strains, shared polyketide synthases (t2PKS, otherKS), NRPS, and genes related to terpene and betalactone biosynthesis are observed (Figure 5A). The presence of siderophore biosynthetic genes suggests a common strategy for iron acquisition. Distinctions, such as lassopeptide genes in cultivated strains and unique distributions of t1PKS1, t3PKS, and lanthipeptide genes in specific uncultivated strains, imply adaptations to specific ecological niches.

## Prediction of lifestyle, nutrition exigence, optimal growth temperature and doubling time

The PhenDB web server analysis conclusively determined that both cultured and uncultured genomes of *Frankia* and *Protofrankia* do not exhibit characteristics typical of obligate intracellular symbionts (Table 4). PhenDB predicted an aerobic nature for all strains, consistent with established knowledge for cultured *Frankia*. The GapMind server analysis identified alanine, aspartate, fumarate, glucose-6-P, L-malate, 2-oxoglutarate, pyruvate, succinate, and propionate as optimal carbon sources for the majority of *Frankia* and *Protofrankia* (Supplementary Table S2), aligning with well-established compositions for routine growing media useful for *Frankia* strains (Blom, 1982; Murry et al., 1984; Lopez et al., 1986; Stowers et al., 1986; Gtari et al., 2015). Notably, Microtrait predicted ammonia as the mineral nitrogen source, excluding nitrite and nitrate, consistent with findings by Blom (1982) and Shipton and Burggraaf (1982). Additionally, GapMind predicted no auxotrophy for any amino acids (Supplementary Table S3).

The Microtrait pipeline predicted distinct temperature preferences and growth rates among cultured and uncultured *Frankia* and *Protofrankia* strains (Figures 4B, 5B). Cultured *Frankia* strains (ACN14a, Ag45/Mut15, Agncl-4) prefer temperatures around 28–29°C. Diverse temperature adaptations are observed in cultured CcI3, CpI1, and QA3, with optimal temperatures of 34.78, 28.75, and 28.85°C, respectively. In contrast, uncultured strains (AgTrS, AiOr, AvVan) exhibit a slightly higher temperature preference at 31–32°C. Uncultured *Frankia* strains UmASH1 and UmASt1, along with most *Protofrankia* strains, lean toward even higher temperatures, around 32.55 and 33.34°C, showcasing variability in temperature preferences.

These optimal temperature values, ranging from 28°C to 35.49°C, generally align with experimentally determined values (Burggraaf and Shipton, 1982; Gtari et al., 2015). For example, the optimal temperature for CpI1, predicted here at 28.75°C, was experimentally determined to be between 30°C and 35°C (Burggraaf and Shipton, 1982). Additionally, strain Agncl-4, predicted here at 28°C, grew optimally between 25°C and 37°C (Nouioui et al., 2023). *Casuarina* isolates, including *Frankia casuarinea* CcI3, which was predicted here at 34.78°C, exhibited maximum growth between 25°C and 37°C (Sayed et al., 1997).

The growth rates among *Frankia* strains vary significantly (Figure 4C). Ag45/Mut15 displays the fastest growth with a doubling time of 3.26 days, followed closely by ACN14a and AgTrS at 4.35 and 3.65 days, respectively. CpI1, UmASt1, UmASH1, and QA3 exhibit moderate growth rates, ranging from 4.75 to 5.48 days. AvVan and CcI3 have slightly longer doubling times at 6.18 and 5.23 days. Notably, Agncl-4 stands out with the slowest growth, displaying a doubling time of 9.60 days. For *Protofrankia*, BMG5.30 is an efficient grower with a short doubling time of 4.60 days (Figure 5C). Cm1Cmnod and Cv1Ctnod1 also show efficient growth (4.91 and 6.64 days) while Dg1 and Dg2 fall into the category of moderate growers (5.04 and 6.75 days). In contrast, Cppng1 and Cppng1Canod exhibit longer doubling times at 21.82 and 21.98 days. The predicted doubling times in this study are consistent with previously experimentally determined values for *Frankia* strain ArI3 (3.8 days, Ringø et al., 1995) and *Frankia torreyi* CpI1 (4.03 days, Burggraaf and Shipton, 1983). However, the experimentally determined doubling time for *Frankia casuarinae* CcI3, which ranges from 1 to 2 days depending on the medium and incubation conditions (Zhongze et al., 1986; Huang and Benson, 2012), is notably shorter than the predicted values obtained in the present study.

## Conclusion and perspectives

Our in-depth analysis explores the cultivability potential of previously deemed unculturable *Frankia* and *Protofrankia* strains. Despite challenges posed by intracellular lifestyle, genomic intricacies, and ecological adaptations, our findings highlight potential cultivability traits. Genomic comparisons reveal differences, offering insights into the ongoing evolutionary changes and adaptations shaped by environmental pressures and biological interactions. Pangenome analysis unveils substantial diversity, suggesting untapped genetic resources for cultivation. Shared metabolic strategies in cellular components, central metabolic pathways, and resource acquisition traits present promising avenues for future cultivation attempts. Stress tolerance mechanisms demonstrate resilience, indicating adaptability to controlled conditions. Secondary metabolite clusters unveil potential ecological competitiveness with potential predisposition to asymbiotic growth in axenic conditions. This study offers a nuanced perspective, highlighting potential breakthroughs in tailored media formulations and optimal growth conditions. Future efforts aimed at cultivating as-yet-uncultured *Frankia* and *Protofrankia* under axenic conditions should prioritize using a mineral growth medium enriched with simple carbon sources like alanine, aspartate, fumarate, glucose-6-P, L-malate, 2-oxoglutarate, pyruvate, succinate, and propionate. These should be provided in aerobic environments, with atmospheric nitrogen being optionally sufficient to minimize contamination risks, though ammonia can be included as a preferred alternative nitrogen source. Temperature adjustments should align with strain preferences: cultured *Frankia* strains thrive at 28–29°C, while *Protofrankia* strains prefer slightly higher temperatures of 32–35°C, maintaining an alkaline pH range for the latter. Given the potential for extended incubation periods required for growth (with predicted doubling times ranging from 3.26 to 9.60 days, but possibly reaching up to 21.98 days), patience is essential, and meticulous monitoring for contaminants is crucial to optimize cultivation conditions.

Recent advancements in microbial technologies have indeed revolutionized our understanding of the challenges associated with yet uncultured microorganisms. Advanced computational tools now enable the extraction of mechanistic insights that influence how microorganisms adapt to laboratory conditions. However, despite the substantial benefits offered by genomics, it requires meticulous consideration of potential biases and errors. Issues such as assembly errors, annotation discrepancies, and variations in sequencing depth can distort gene content and complicate the interpretation of data (Stein, 2001; Quince et al., 2017; Tam et al., 2019). These factors are particularly critical in studies utilizing comparative genomics or downstream applications. MAGs encounter additional obstacles, including gaps resulting from low-read depths (Chu et al., 2019), fragmentation due to strain divergence (Chen et al., 2020), and assembly errors like repeat collapse and chimeric reads (Alneberg et al., 2014; Olson et al., 2019; Mineeva et al., 2020). Short scaffolds can lead to binning errors and the presence of unreliable MAGs contaminated by phage or plasmid fragments (Chen et al., 2020). These limitations underscore the critical importance of rigorous quality assessment in the interpretation of MAG data, particularly within the fields of microbiological and environmental genomics.

## Data availability statement

The original contributions presented in the study are included in the article/Supplementary material, further inquiries can be directed to the corresponding author.

## Author contributions

MG: Conceptualization, Investigation, Software, Writing – original draft, Writing – review & editing. RM: Formal analysis, Investigation, Software, Writing – review & editing. FG-G: Investigation, Software, Writing – review & editing. KB: Funding acquisition, Writing – review & editing. IS: Investigation, Software, Writing – review & editing.

## Footnotes

^1^ http://phendb.org/
