# Supplementary material for: MAGs-centric crack: how long will, spore-positive Frankia and most Protofrankia, microsymbionts remain recalcitrant to axenic growth?
Source: Front Microbiol. 2024 Jul 31;15:1367490. doi: 10.3389/fmicb.2024.1367490 (PMC11323853; doi:10.3389/fmicb.2024.1367490)
Supplement: Supplementary file 2 [file Data_Sheet_1.PDF]

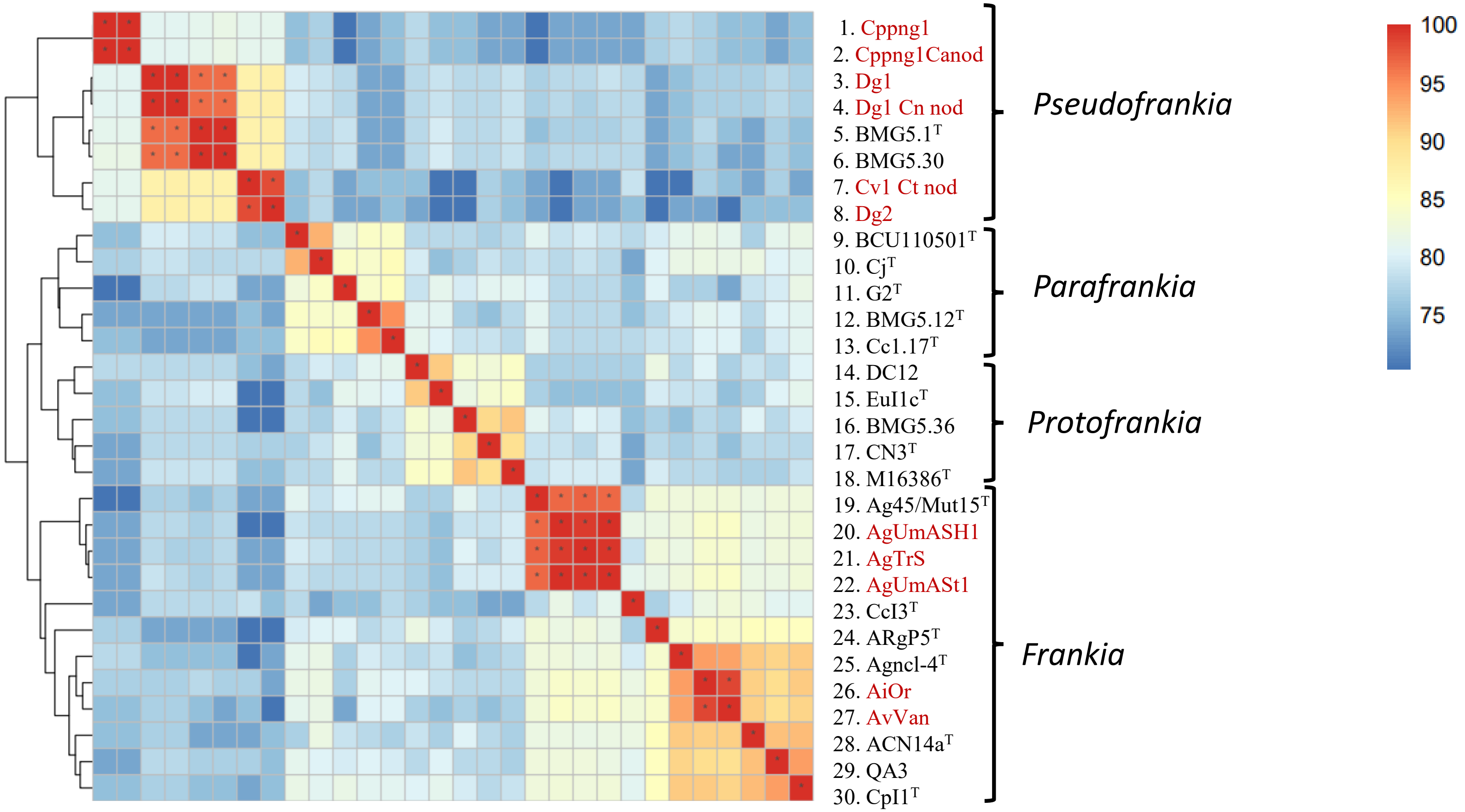

Fig. S1 : Clustering of of members of the *Frankiaceae* family based on small MASH distance. Uncultivable strains are indicated in red.

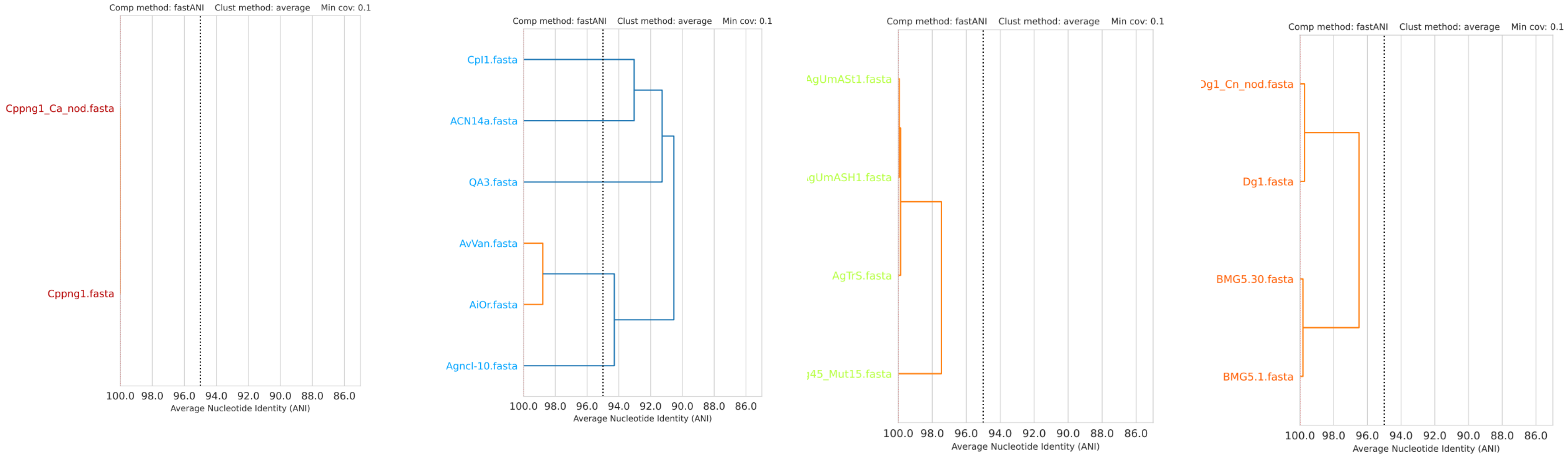

Fig. S2 : Average nucleotide identity (ANI) for uncultured *Frankia* and *Protofrankia* and their closely related cultured conterparts.

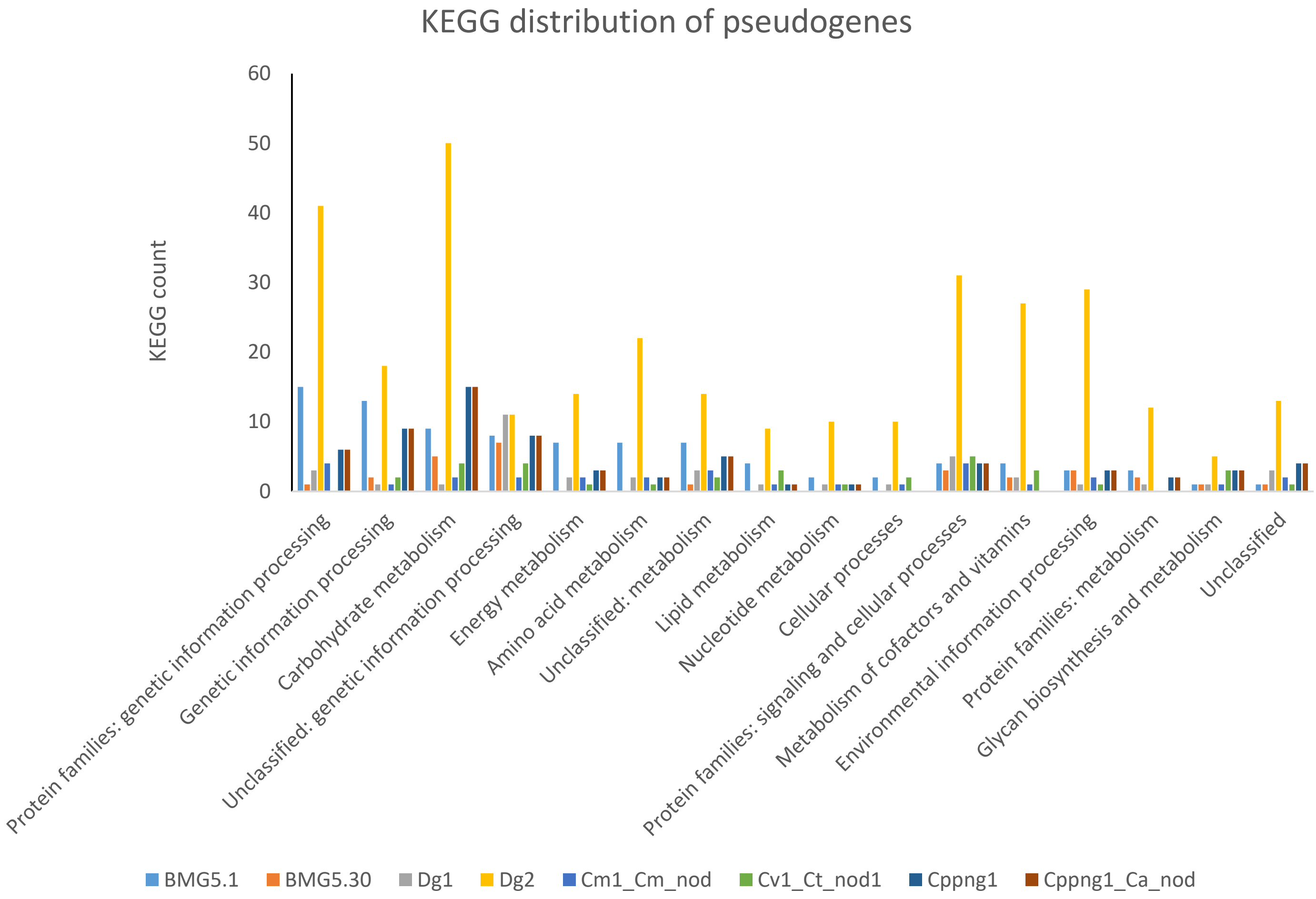

Fig. S3 : KEGG distribution of pseudogenes in *Protofrankia* genomes

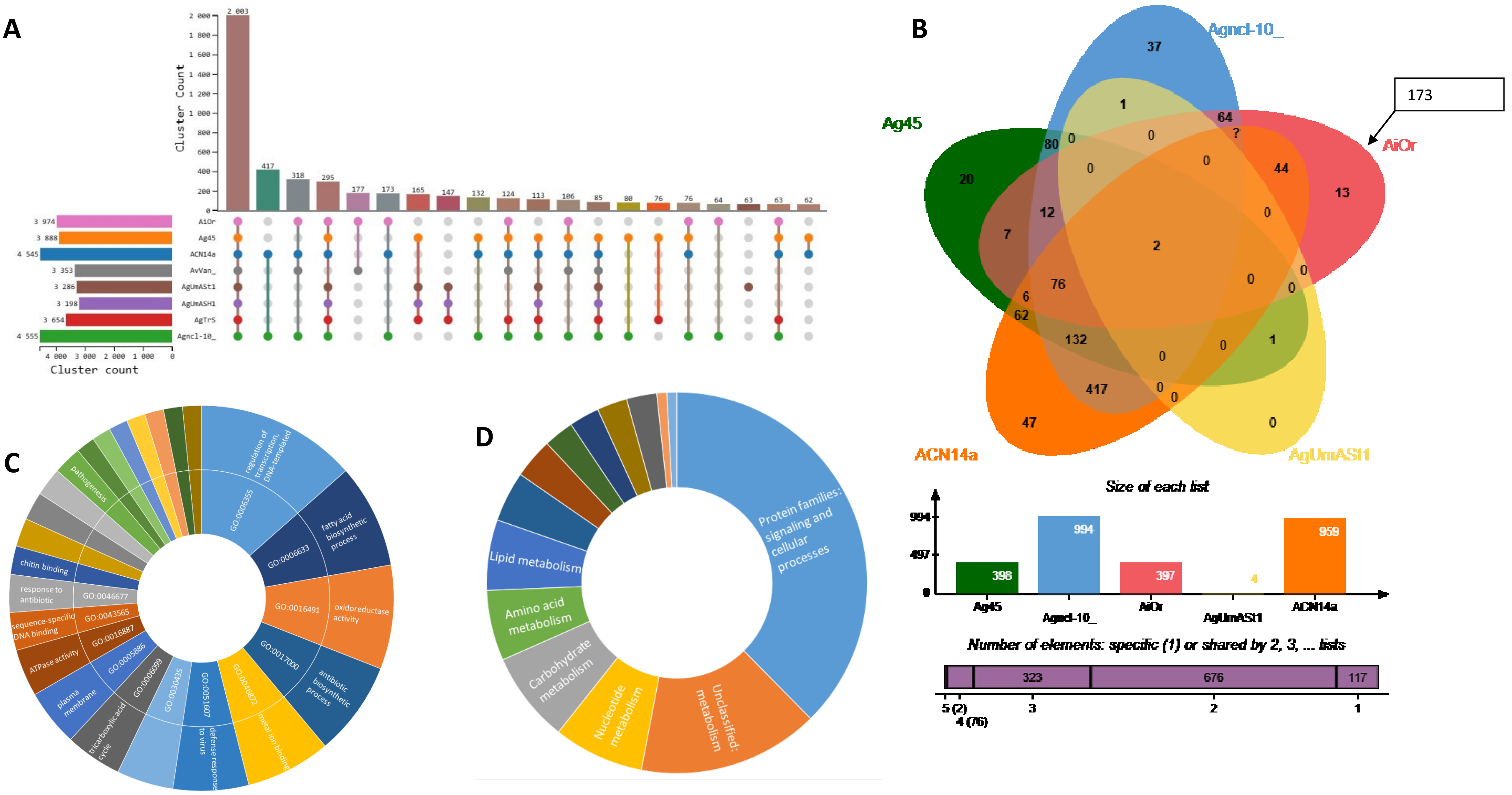

Fig. S4: OrthoVenn3 analysis depicting the cluster count for each genome among uncultured *Frankia* and their closely related counterparts, illustrating shared and unique clusters (A). Venn diagram showcasing shared and unique gene clusters (B). Gene Ontology (GO) enrichment (C) and KEGG distribution (D) of the 132 GOs (521 genes) absent in uncultured *Frankia*.

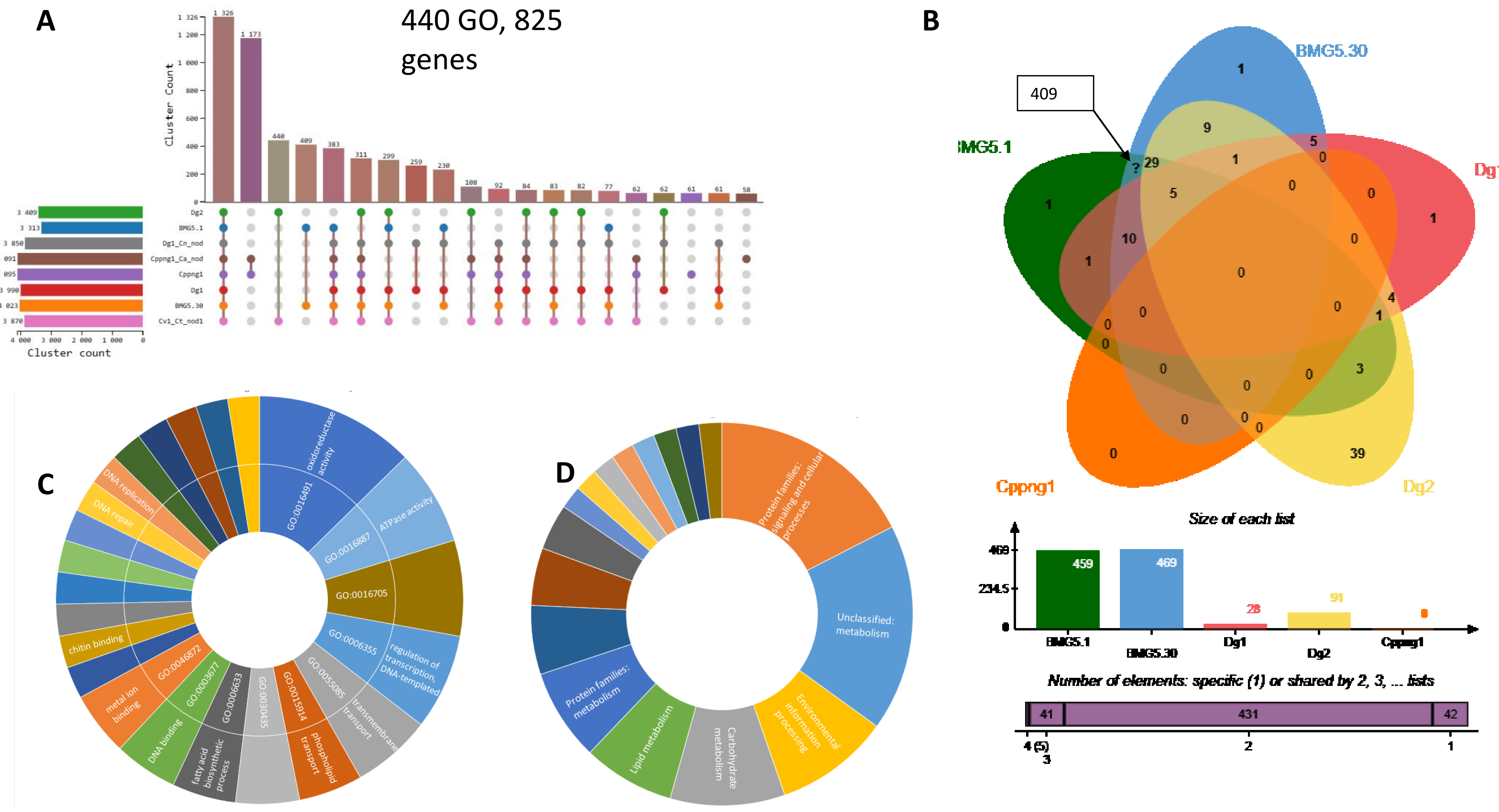

Fig. S5: OrthoVenn3 analysis depicting the cluster count for each genome among uncultured *Protofrankia* and their closely related counterparts, illustrating shared and unique clusters (A). Venn diagram showcasing shared and unique gene clusters (B). Gene Ontology (GO) enrichment (C) and KEGG distribution (D) of the 440 GOs (825 genes) absent in uncultured *Protofrankia*.

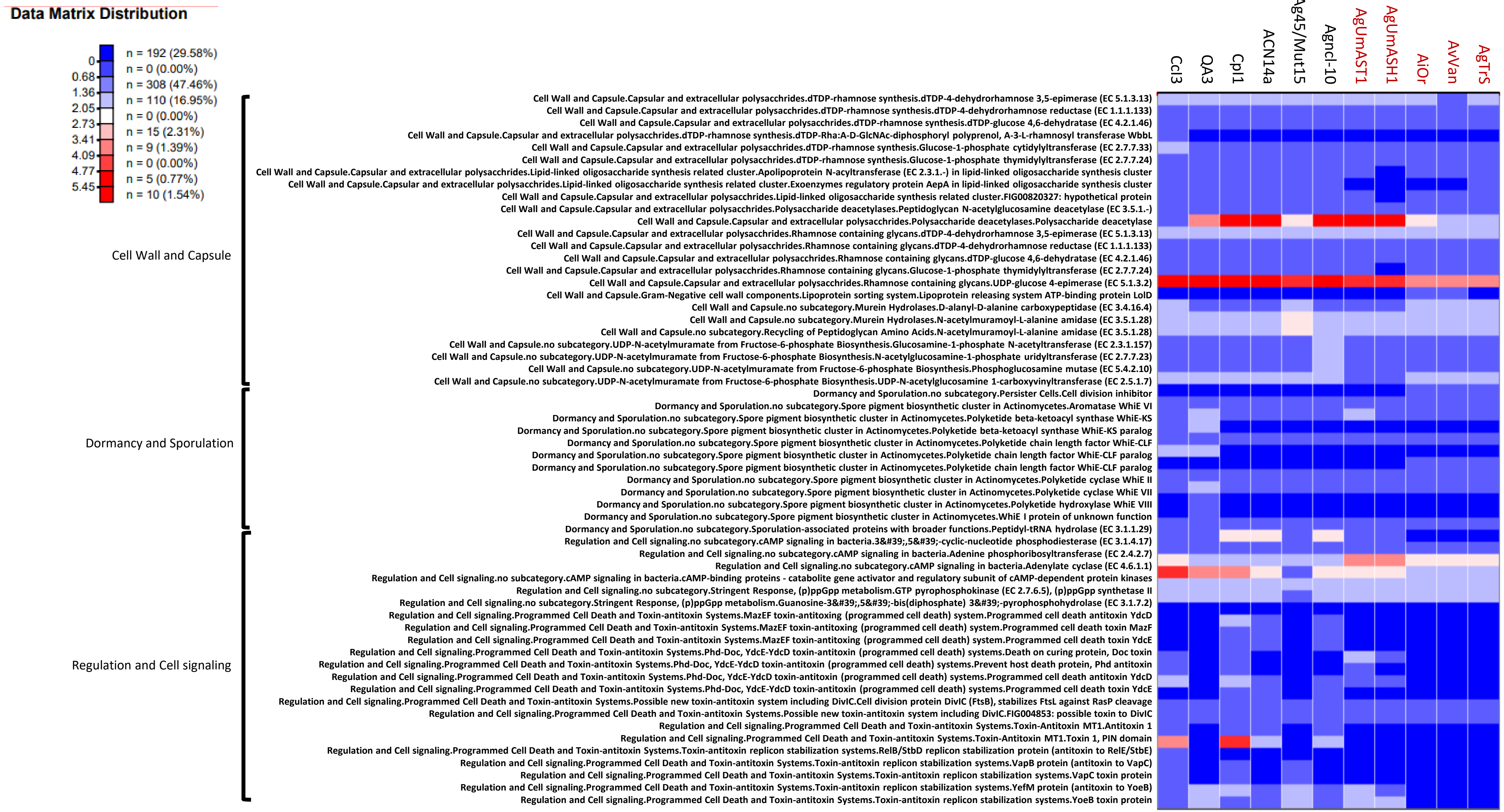

Fig. S6: Comparative heatmap analysis of 'Cell Wall and Capsule,' 'Dormancy and Sporulation,' and 'Regulation and Cell Signaling' categories between uncultured *Frankia* and their respective closely related counterparts. Uncultivable strains are indicated in red.

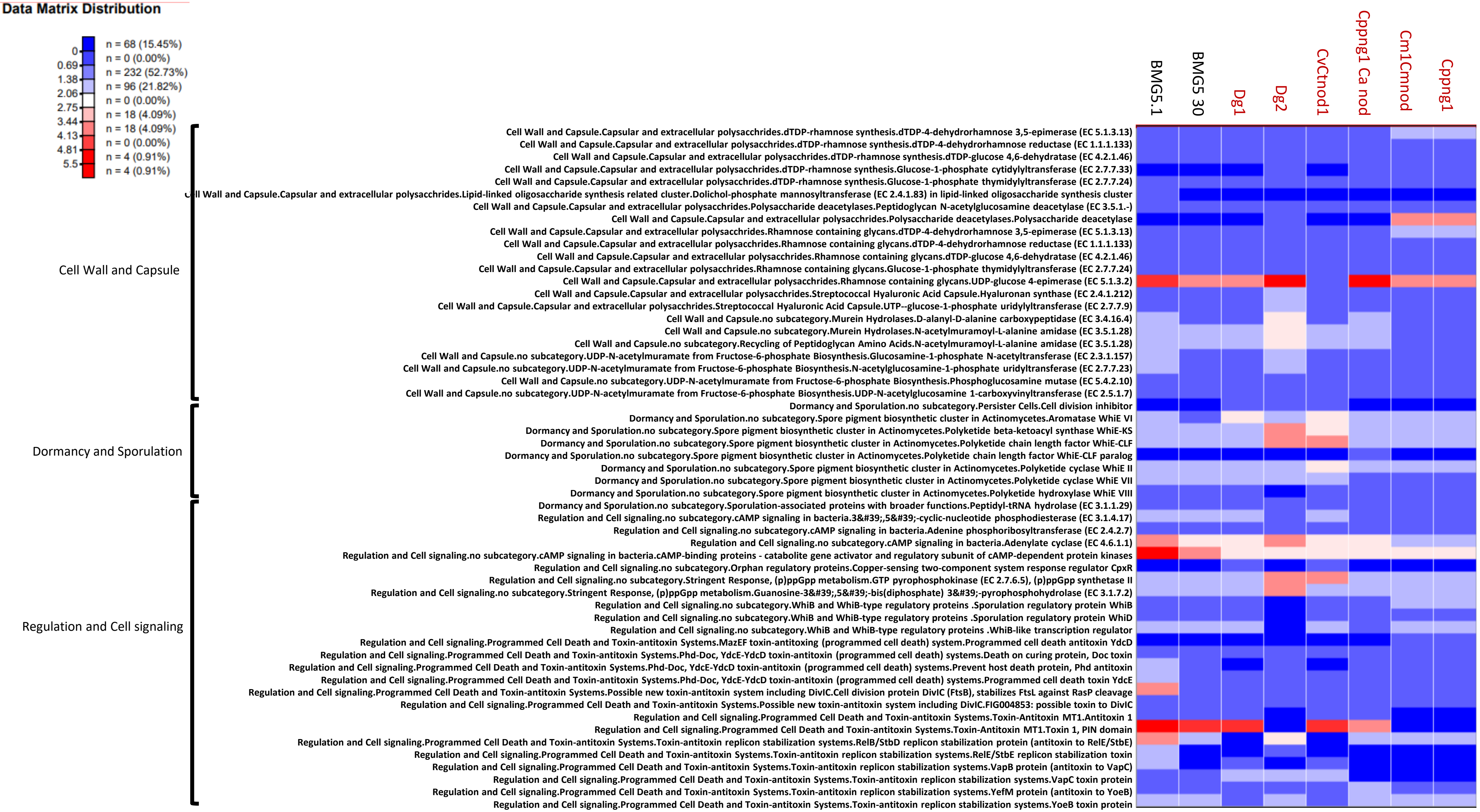

Fig. S7: Comparative heatmap analysis of 'Cell Wall and Capsule,' 'Dormancy and Sporulation,' and 'Regulation and Cell Signaling' categories between uncultured *Protofrankia* and their respective closely related counterparts. Uncultivable strains are indicated in red.

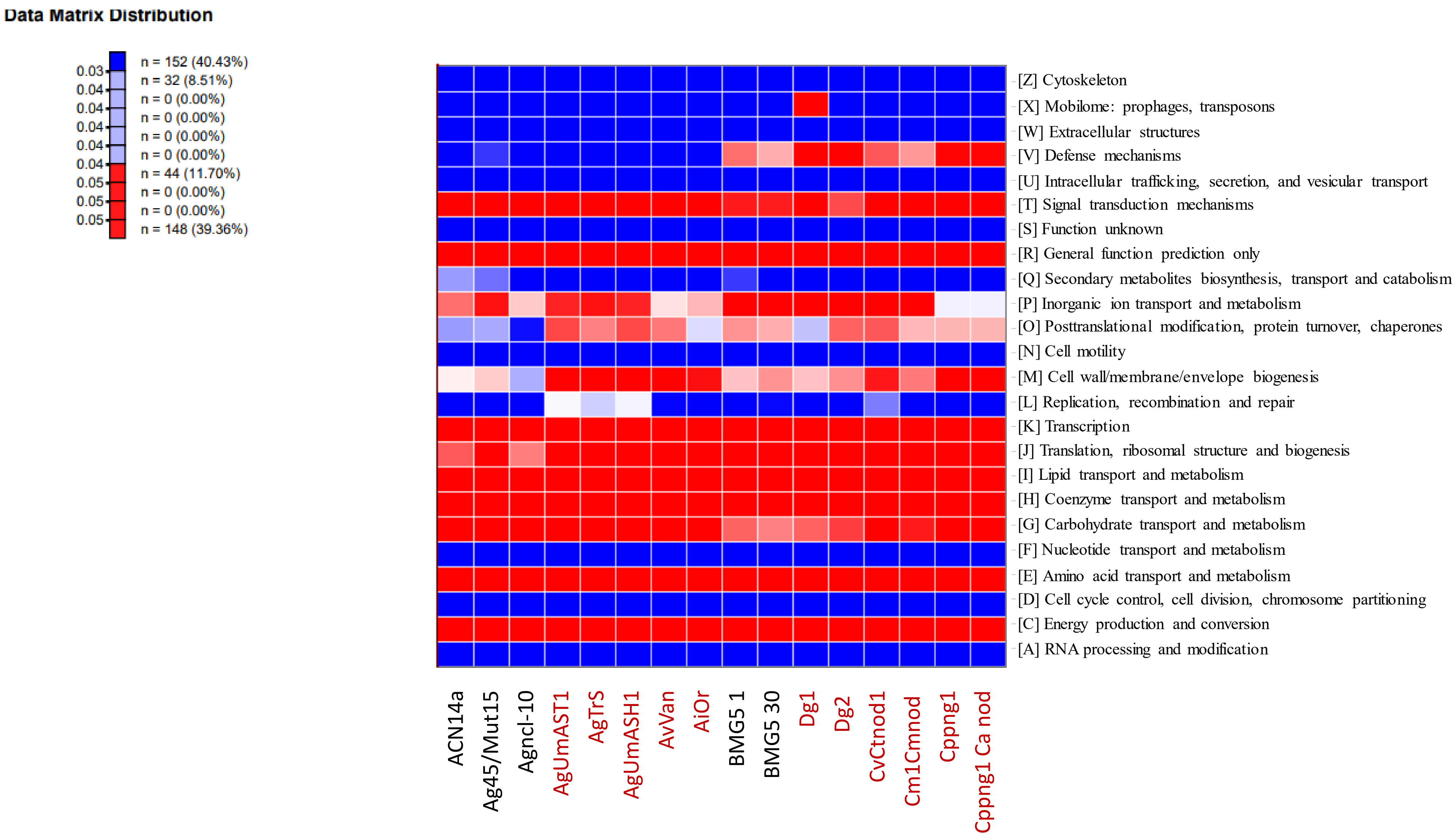

Fig. S8: Comparative heatmap analysis of COG annotations for uncultured *Frankia* and *Protofrankia*, alongside their closely related counterparts. Completeness of the pathways may be assessed with closely related and particularly complete genomes such as ACN14a and Dg1. Uncultivable strains are indicated in red.

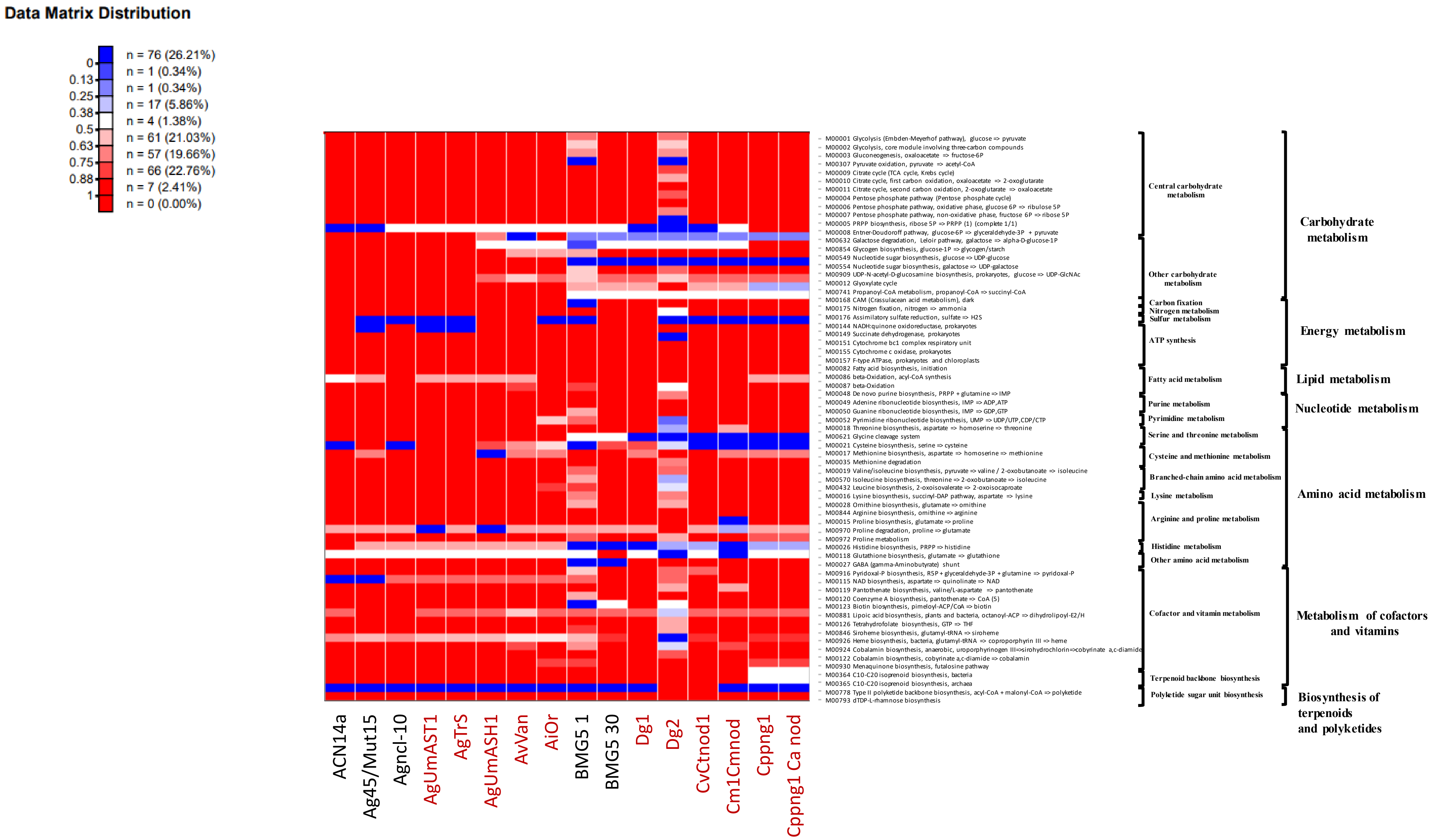

Fig. S9: Heatmap comparison of KEGG annotations between uncultured *Frankia* and *Protofrankia*, along with their closely related counterparts. Completeness of the pathways may be assessed with closely related and particularly complete genomes such as ACN14a and Dg1. Uncultivable strains are indicated in red.
